# Chronic Kidney Disease Severity and Risk of Cognitive Impairment

**DOI:** 10.1001/jamanetworkopen.2025.59834

**Published:** 2026-02-17

**Authors:** Zhijie Huang, Kristine Yaffe, Changwei Li, Cissy Xiao, Yang Pan, Xiao Sun, Amanda H. Anderson, Jiang He, Bernard G. Jaar, Heedeok Han, Krzysztof Kiryluk, Mahboob Rahman, Panduranga Rao, Ana C. Ricardo, Vallabh O. Shah, Anand Srivastava, Jonathan J. Taliercio, Manjula Kurella Tamura, Mark L. Unruh, Matthew R. Weir, James P. Lash, Lydia A. Bazzano, Jing Chen, Katherine T. Mills, Tanika N. Kelly

**Affiliations:** 1 Department of Epidemiology, Tulane University School of Public Health and Tropical Medicine, New Orleans, Louisiana; 2 Division of Endocrinology, Diabetes, and Metabolism, Department of Medicine, College of Medicine, University of Illinois Chicago, Chicago; 3 Department of Psychiatry, University of California, San Francisco; 4 Department of Neurology, University of California, San Francisco; 5 Department of Epidemiology and Biostatistics, University of California, San Francisco; 6 Department of Epidemiology, Peter O’Donnell Jr. School of Public Health at UT Southwestern Medical Center, Dallas, Texas; 7 Department of Epidemiology, University of Alabama at Birmingham School of Public Health, Birmingham; 8 Division of Nephrology, Department of Medicine, Johns Hopkins University, Baltimore, Maryland; 9 Division of Nephrology, Department of Medicine, Columbia University, New York, New York; 10 Division of Nephrology and Hypertension, University Hospitals Cleveland Medical Center, Cleveland, Ohio; 11 Division of Nephrology, Department of Medicine, University of Michigan, Ann Arbor; 12 Division of Nephrology, Department of Medicine, College of Medicine, University of Illinois Chicago, Chicago; 13 Department of Internal Medicine and Biochemistry, University of New Mexico, Albuquerque; 14 Department of Kidney Medicine, Cleveland Clinic, Cleveland, Ohio; 15 Division of Nephrology, Department of Medicine, Stanford University School of Medicine, Palo Alto, California; 16 Division of Nephrology, Department of Internal Medicine, University of New Mexico, Albuquerque; 17 Division of Nephrology, Department of Medicine, University of Maryland School of Medicine, Baltimore; 18 Department of Medicine, O’Donnell Jr School of Public Health, UT Southwestern Medical Center, Dallas, Texas; 19 Department of Epidemiology, O’Donnell Jr School of Public Health, UT Southwestern Medical Center, Dallas, Texas

## Abstract

**Importance:**

Associations between chronic kidney disease (CKD) severity and incident cognitive impairment have not been evaluated in a cohort of patients with CKD.

**Objective:**

To investigate associations between CKD severity, based on the estimated glomerular filtration rate (eGFR) and urinary protein to creatinine ratio (UPCR), and incident cognitive impairment in a cohort of patients with CKD.

**Design, Setting, and Participants:**

This cohort study investigated 5607 participants with CKD from the ongoing Chronic Renal Insufficiency Cohort (CRIC) Study who were enrolled between 2003 to 2008 and 2013 to 2015. Statistical analysis was conducted from August 2024 to December 2025.

**Exposure:**

Estimated glomerular filtration rate and UPCR.

**Main Outcomes and Measures:**

Global cognition and domains of verbal memory and delayed recall, attention and processing speed, and executive function were evaluated using the Modified Mini-Mental Status Examination, Buschke Selective Reminding test, and Trail Making Tests A and B, respectively. For each test, impairment was defined as a score at least 1 SD worse than the baseline cohort mean. After those with cognitive impairment at baseline were excluded, Cox proportional hazards regression models tested associations of baseline eGFR and UPCR, individually and together, with time to cognitive impairment after adjusting for demographic, lifestyle, and clinical risk factors.

**Results:**

The 5607 CRIC participants included 3159 men (56.3%), the mean (SD) cohort age was 59.6 (10.8) years at baseline, and the median follow-up was 6 years (range, 0.5-16 years) for the Modified Mini-Mental State Examination, 4 years (range, 0.5-13 years) for the Buschke Selective Reminding Test, and 4 years (range, 0.5-13 years) for Trail Making Tests A and B. In multivariable-adjusted analyses, each 1 SD higher log-transformed UPCR was associated with 21% increased risk of impairments in attention and processing speed (hazard ratio [HR], 1.21; 95% CI, 1.05-1.41; *P* = .01) and 16% increased risk of impairment in executive function (HR, 1.16; 95% CI, 1.02-1.31; *P* = .02). Each 1 SD lower eGFR was associated with 21% increased risk of impairment in attention and processing speed (HR, 1.21; 95% CI, 1.05-1.38; *P* = .006). Findings for UPCR remained nominally significant after further adjustment for eGFR, while eGFR findings were attenuated on adjustment for UPCR. Patients with a combined eGFR less than 60 mL/min/1.73 m^2^ and UPCR of 150 mg/g or more had a significant 38% increased risk of impairment in global cognition (HR, 1.38; 95% CI, 1.05-1.82; *P* = .003) compared with those with an eGFR of 60 mL/min/1.74 m^2^ or more and UPCR less than 150 mg/g.

**Conclusions and Relevance:**

This cohort study of patients with CKD suggests that a more advanced CKD stage was associated with increased incidence of cognitive impairment. These findings underscore CKD severity as a risk factor for cognitive decline across the CKD spectrum.

## Key Points

**Question:**

Is chronic kidney disease (CKD) severity associated with incident cognitive impairment among patients with CKD?

**Findings:**

In this cohort study of 5607 participants with CKD, a higher urinary protein to creatinine ratio was associated with impairments in attention and processing speed as well as executive function, while a lower estimated glomerular filtration rate (eGFR) was not associated with any cognitive impairment end points after adjusting for the urinary protein to creatinine ratio. The combination of a higher urinary protein to creatinine ratio and a lower eGFR was associated with impairments in global cognition.

**Meaning:**

This study suggests that a more advanced CKD stage is associated with cognitive impairment.

## Teaser

This cohort study of participants in the Chronic Renal Insufficiency Cohort investigates whether chronic kidney disease severity is associated with incident cognitive impairment.

## Introduction

The global burden of dementia is substantial, affecting approximately 57.4 million adults in 2019 and ranking among the top 10 leading causes of mortality.^1,2^ Previous prospective studies have identified chronic kidney disease (CKD) as an independent risk factor for incident dementia.^3,4,5^ Likewise, CKD has been prospectively associated with key dementia risk factors and symptoms, including cognitive decline and cognitive impairment based on repeated cognitive testing.^6,7^ Among adults with CKD, cross-sectional studies have shown higher frequencies of cognitive impairment with more advanced disease.^8,9^ These data suggest that risks of cognitive decline may vary markedly across the spectrum of CKD severity. However, only a few population-based studies have prospectively examined kidney function across the CKD range with cognitive phenotypes,^4,5,7,10^ and fewer evaluated both the estimated glomerular filtration rate (eGFR) and urinary protein.^5,11^ Among studies that have investigated both measures of kidney function, urinary protein has been more consistently associated with dementia^5,11^ and cognitive impairment^10^ than eGFR. Given the close link between proteinuria and endothelial vascular function, proteinuria might better reflect small vessel disease in the brain and resulting cognitive decline.^12^ Further research evaluating both eGFR and proteinuria in association with cognition phenotypes is warranted.

The present study investigated the prospective associations between CKD severity, based on eGFR and urinary protein to creatinine ratio (UPCR), and incident cognitive impairment among patients with CKD from the Chronic Renal Insufficiency Cohort (CRIC) Study. We hypothesized that a lower eGFR and a higher UPCR would be associated with increased incidence of cognitive impairment.

## Methods

### Study Population

**Figure 1.**
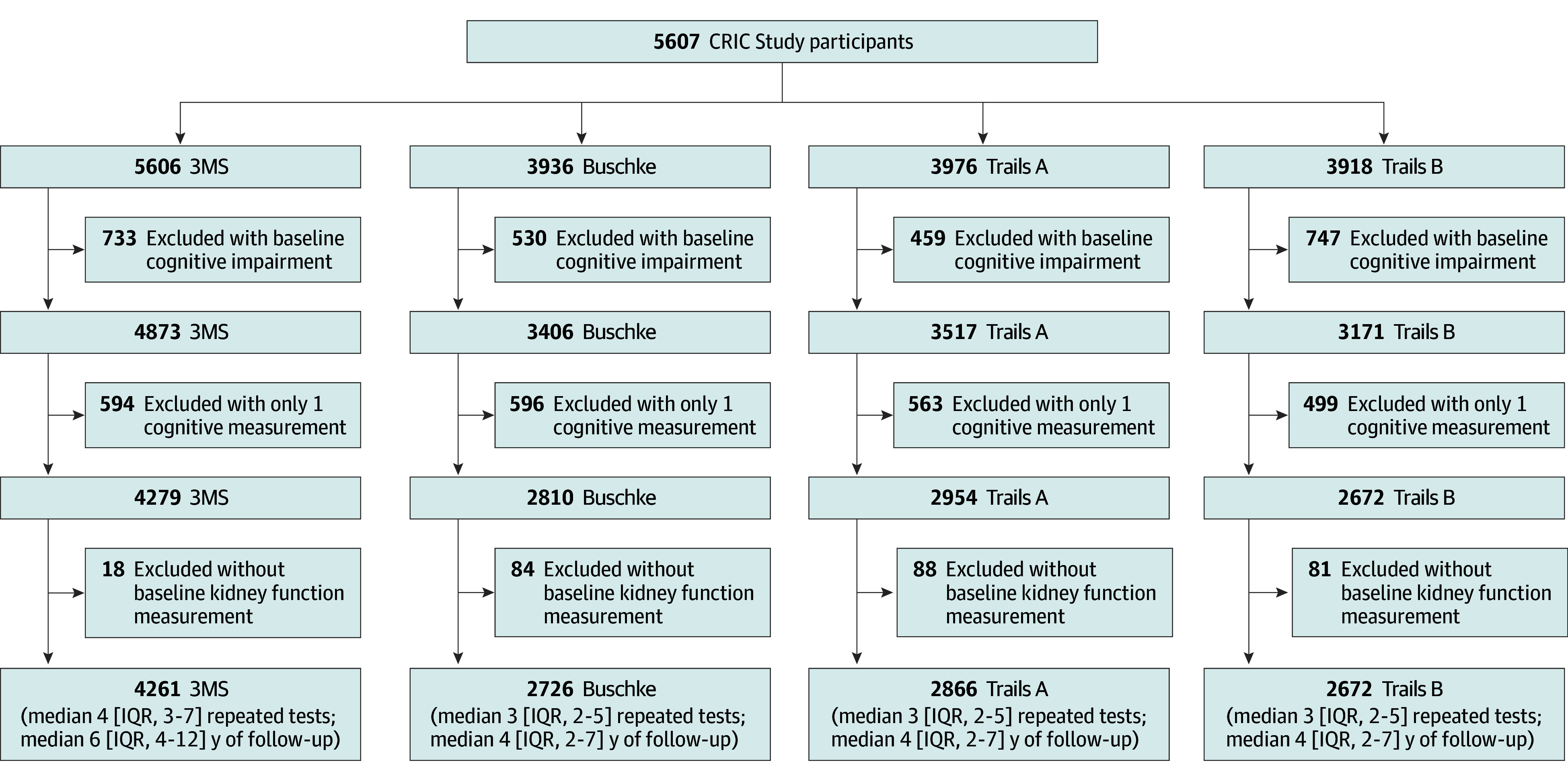
Flowchart of Chronic Renal Insufficiency Cohort (CRIC) Study Participant Inclusion 3MS indicates Modified Mini-Mental State Examination; Buschke, Buschke Selective Reminding Test; Trails A, Trail Making Test A; and Trails B, Trail Making Test B.

The CRIC Study is an ongoing prospective cohort study designed to identify risk factors for the progression of CKD and cardiovascular disease in the setting of CKD.^13^ Between 2003 to 2008 and 2013 to 2015, the CRIC Study has enrolled a diverse sample of 5607 adults aged 21 to 79 years across a broad spectrum of kidney disease severity from 7 clinical centers (eMethods in Supplement 1). The present study included participants free of cognitive impairment at baseline, as assessed by the Modified Mini-Mental State Examination (n = 4261), Buschke Selective Reminding Test (n = 2726), Trail Making Test A (n = 2866), and Trail Making Test B (n = 2591), with a median follow-up of 6 years (range, 0.5-16 years) for the Modified Mini-Mental State Examination, 4 years (range, 0.5-13 years) for the Buschke Selective Reminding Test, 4 years (range, 0.5-13 years) for Trail Making Test A, and 4 years (range, 0.5-13) years for Trail Making Test B. A flowchart of participants is shown in Figure 1. Institutional review boards at all participating institutions (University of Pennsylvania; John Hopkins University/University of Maryland; Case Western Reserve University; University of Michigan at Ann Arbor; University of Illinois at Chicago; Kaiser Permanente of Northern California/University of California, San Francisco; and Tulane University) approved the study protocol, and participants provided written informed consent. The study followed the Strengthening the Reporting of Observational Studies in Epidemiology (STROBE) reporting guideline for reporting cohort studies.

### Exposure Assessment

Fasting blood samples were obtained at the baseline examination and used for the measurement of serum creatinine and cystatin C (eMethods in Supplement 1). The eGFR was calculated using the race-neutral 2021 Chronic Kidney Disease Epidemiology Collaboration (CKD-EPI) creatinine-cystatin C equation.^14^ Due to the limited availability of urinary albumin to creatinine ratio (UACR) data, we examined the UPCR in our primary analyses, which was estimated from a 24-hour urine test and spot sample measures when the 24-hour test was unavailable (<1% of the sample). The eGFR was categorized as G1-G2 (≥60 mL/min/1.73 m^2^), G3a (45-59 mL/min/1.73 m^2^), G3b (30-44 mL/min/1.73 m^2^), and G4-G5 (<30 mL/min/1.73 m^2^).^15^ The UPCR was categorized as normal to mildly increased (P1; <150 mg/g), moderately increased (P2; 150-500 mg/g), and severely increased (P3; >500 mg/g), with UPCR thresholds selected to maximize correlations with standard albuminuria staging.^16,17,18^ To evaluate eGFR and UPCR jointly, participants were categorized into 4 groups: G1-G2/P1 (reference), G1-G2/P2-P3, G3-G5/P1, and G3-G5/P2-P3. Broader groupings were used to handle smaller cell sizes in the joint analyses. In sensitivity analyses using the subsample with UACR measures, the UACR was categorized as normal to mildly increased (A1; <30 mg/g), moderately increased (A2; 30-300 mg/g), and severely increased (A3; >300 mg/g).

### Covariates

Baseline information on age, sex, race and ethnicity (Hispanic, non-Hispanic Black, non-Hispanic White, and other [American Indian or Alaskan Native, Asian or Asian American, or Native Hawaiian or Other Pacific Islander]), educational level, smoking status, alcohol intake, physical activity, medications, medical history, and depression was collected by validated questionnaires. Because race and ethnicity have been associated with both CKD and cognitive decline, these variables were collected to minimize confounding in the current study. Blood pressure, height, and weight were measured by physical examination following validated protocols (eMethods in Supplement 1).

### Cognitive Function Assessment

Global cognitive function has been assessed annually or biennially in the CRIC since 2003 using the Modified Mini-Mental State Examination. Introduced to the entire CRIC in 2008, the Buschke Selective Reminding Test, Trail Making Test A, and Trail Making Test B were administered annually or biennially to assess domains of verbal memory and delayed recall, attention and processing speed, and executive function. More details about each cognitive test are included in the eMethods in Supplement 1. Similar to previous CRIC reports,^19,20,21^ incident cognitive impairment was defined for each test as a score at least 1 SD worse than the cohort mean at baseline. Those with cognitive impairment at baseline were excluded from longitudinal analyses. To assess the robustness of our findings to alternate definitions of cognitive impairment, we further defined cognitive impairment as a Modified Mini-Mental State Examination score of less than 80, which has been used in several previous reports,^22,23^ and by a test score at least 5% worse than the baseline score, which reflects meaningful cognitive change.^24^

### Statistical Analysis

Statistical analysis was conducted from August 2024 to December 2025. Characteristics of study participants at baseline were summarized as numbers and percentages for categorical variables and either mean (SD) or median (IQR) vallues for continuous variables. After excluding those with cognitive impairment at baseline, associations of eGFR, UPCR, and joint eGFR and UPCR with incident cognitive impairment were assessed using 3 Cox proportional hazards regression models. Model 1 adjusted for demographic variables, clinical center, and baseline cognitive score. Model 2 additionally adjusted for lifestyle and behavioral risk factors. The fully adjusted model, model 3, further included clinical variables (eMethods in Supplement 1). To assess their relative importance, models further adjusted for UPCR when evaluating eGFR, and vice versa. Interactions between eGFR and UPCR were investigated by including a product term in models with both variables.

With the use of a continuous eGFR and a log-transformed UPCR, restricted cubic splines were used to investigate nonlinear associations with incident cognitive impairment in our fully adjusted model. Nonlinearity was assessed by evaluating *P* values from likelihood ratio tests comparing models with both restricted spline and linear terms with models with only a linear term. In the absence of a nonlinear association, *P* values for linear associations from the models without the restricted spline terms were presented. A default of 3 knots was used for both restricted cubic splines. Furthermore, to evaluate associations of eGFR and UPCR with longitudinal changes in cognitive test scores, we implemented latent process mixed models with a beta link for each cognitive test (eMethods in Supplement 1). These models account for the discrete and curvilinearity properties of psychometric tests, including ceiling and floor effects.^25,26,27^

Subgroup analyses tested associations of the continuous eGFR and log UPCR measures with cognitive impairment according to age, sex, race and ethnicity, and diabetes status. Sensitivity analyses used the 2021 race-neutral creatinine and cystatin C CRIC equation^28^ instead of the race-neutral CKD-EPI equation. UPCR analyses were compared with UACR among the subset of participants with available data. Cognitive impairment end points defined by an incident score of less than 80 on the Modified Mini-Mental State Examination and 5% worsening in each test score from baseline^24^ (equivalent to a 5-point decrease for the Modified Mini-Mental State Examination, a 1-point decrease for the Buschke Selective Reminding Test, and 15-point increases for Trail Making Tests A and B) were further evaluated in sensitivity analyses. Spline analyses with 4 and 5 knots were also examined. In addition, sensitivity analyses accounting for the competing risks of death using Fine-Gray subdistribution hazards models were also conducted.

To account for testing 4 cognitive end points, a false discovery rate (FDR) correction was used for all analyses. Restricted cubic spline analysis was conducted using the lgtphcurv9 Macro with SAS, version 9.4 (SAS Institute Inc), while other analyses were performed using R, version 4.0.2 (R Project for Statistical Computing). All *P* values were from 2-sided tests and results were deemed statistically significant at *P* < .05.

## Results

### Baseline Characteristics

The characteristics of the 5607 CRIC participants (mean [SD] age, 59.6 [10.8] years; 3159 men [56.3%] and 2448 women [43.7%]; 732 Hispanic [12.9%], 2415 non-Hispanic Black [43.1%], 2272 non-Hispanic White [40.5%]; and 197 other race or ethnicity [3.5%]) who were included in 1 or more of the cognition analyses are shown in Table 1 and eTable 1 in Supplement 1. As expected in a CKD cohort, there was a high frequency of hypertension (4849 [86.5%]) and self-reported cardiovascular disease (1876 [33.5%]). Likewise, the mean (SD) eGFR was low, at 52.3 (19.6) mL/min/1.73 m^2^, and the median UPCR was high, at 153 mg/g (IQR, 58-682 mg/g).

**Table 1.** Characteristics of CRIC Study Participants at Their First Cognitive Assessment

Characteristic	CRIC cohort (N = 5607)
Age, mean (SD), y	59.6 (10.8)
Sex, No. (%)	
Male	3159 (56.3)
Female	2448 (43.7)
Race and ethnicity, No. (%)	
Hispanic	723 (12.9)
Non-Hispanic Black	2415 (43.1)
Non-Hispanic White	2272 (40.5)
Other^a^	197 (3.5)
Educational level, No. (%)	
Less than high school	1135 (20.2)
High school	1032 (18.4)
Some college	1626 (29.0)
College degree and higher	1812 (32.4)
Missing	2 (0.04)
Depression, No. (%)	894 (16.5)
Missing	185 (3.3)
Current smoker, No. (%)	704 (12.6)
Alcohol use, No. (%)	3459 (61.7)
Physical activity, mean (SD), METs/wk	194.5 (141.9)
Missing, No. (%)	43 (0.8)
BMI, mean (SD)	32.3 (7.6)
Missing, No. (%)	32 (0.6)
Systolic BP, mean (SD), mm Hg	128.5 (21.4)
Missing, No. (%)	5 (0.1)
ACE inhibitor or ARB use, No. (%)	3826 (68.8)
Missing	41 (0.7)
Hypertension, No. (%)	4849 (86.5)
Missing	2 (0.04)
Diabetes, No. (%)	2881 (51.4)
Self-reported cardiovascular disease, No. (%)	1876 (33.5)
White blood cell count, median (IQR), per μL	6.3 (5.2-7.8)
Missing, No. (%)	160 (2.9)
Hemoglobin, mean (SD), g/dL	12.7 (1.8)
Missing, No. (%)	161 (2.9)
eGFR, mean (SD), mL/min/1.73 m^2^	52.3 (19.6)
Missing, No. (%)	29 (0.5)
eGFR category, mL/min/1.73 m^2^, No. (%)	
G1-G2 (≥60)	1822 (32.7)
G3a (45-59)	1528 (27.4)
G3b (30-44)	1500 (26.9)
G4-G5 (<30)	725 (13.0)
Missing	29 (0.5)
UPCR, median (IQR), mg/g	153 (58-682)
Missing, No. (%)	409 (7.3)
UPCR category, mg/g, No. (%)	
No to mild proteinuria (<150)	2578 (49.6)
Moderate proteinuria (150-500)	1097 (21.1)
Severe proteinuria (>500)	1521 (29.3)
Missing	409 (7.3)

Abbreviations: ACE, angiotensin-converting enzyme; ARB, angiotensin receptor blocker; BMI, body mass index (calculated as weight in kilograms divided by height in meters squared); BP, blood pressure; CRIC, Chronic Renal Insufficiency Cohort; eGFR, estimated glomerular filtration rate; MET, metabolic equivalent of task; UPCR, urine protein to creatinine ratio.

SI conversion factors: To convert white blood cell count to 10^9^/L, multiply by 0.001; and hemoglobin to grams per liter, multiply by 10.0.

^a^ American Indian or Alaskan Native, Asian or Asian American, or Native Hawaiian or Other Pacific Islander.

### Prospective Associations Between eGFR and Cognitive Impairment

We observed significant prospective associations between eGFR-based CKD severity and impairment in attention and processing speed (Table 2). In our fully adjusted model, each 1 SD lower baseline eGFR was associated with a 21% increased risk of impairment in attention and processing speed (hazard ratio [HR], 1.21; 95% CI, 1.05-1.38; *P* = .006), which remained significant after FDR correction for multiple testing. Likewise, ordinal categorical analyses showed a graded and nominally significant increase in risk of this cognition end point with more advanced CKD, with stage G4-G5 associated with a 54% increased risk of impairment compared with stage G1-G2 (HR, 1.54; 95% CI, 1.05-2.27; *P* = .03 for linear trend). Spline analyses using continuous eGFR supported linear associations, with global cognitive impairment and impairment in attention and processing speed achieving nominal and FDR significance, with no evidence of nonlinear associations (eFigure 1 in Supplement 1). Likewise, latent process mixed models also identified significant associations of decreasing eGFR with longitudinal declines in global cognition and attention and processing speed, along with executive function (eTable 2 in Supplement 1). In analyses that further adjusted the UPCR (eTable 3 in Supplement 1), associations between eGFR and impairment in attention and processing speed were attenuated and became nonsignificant. No associations between eGFR alone and impairment in verbal memory and delayed recall based on the Buschke Selective Reminding Test and executive function based on the Trail Making Test B were observed.

**Table 2.** Associations Between Baseline eGFR Stage and Incident Cognitive Impairment

Characteristic	No. of patients	Continuous eGFR		HR (95% CI) for eGFR category, mL/min/1.73 m^2^				*P* value
		HR (95% CI)^a^	*P* value	≥60	45-59	30-44	<30	
**Impairment in global cognition (Modified Mini-Mental State Examination)**								
Events, No.	NA	555	NA	129	180	169	77	NA
Person-years	NA	31 529	NA	11 963	8936	7656	2974	NA
Incidence rates per 1000 person-years	NA	17.6	NA	10.8	20.1	22.1	25.9	NA
Model 1^b^	4259	1.20 (1.08-1.33)	<.001^c^	1 [Reference]	1.37 (1.09-1.73)	1.32 (1.04-1.67)	1.54 (1.14-2.08)	.004^c^
Model 2^b^	4106	1.18 (1.06-1.31)	.002^c^	1 [Reference]	1.33 (1.05-1.68)	1.29 (1.01-1.65)	1.48 (1.09-2.01)	.01^c^
Model 3^b^	4058	1.11 (1.00-1.24)	.06	1 [Reference]	1.26 (0.99-1.61)	1.16 (0.90-1.49)	1.26 (0.91-1.74)	.18
**Impairment in verbal memory and delayed recall (Buschke Selective Reminding Test)**								
Events, No.	NA	365	NA	98	105	96	66	NA
Person-years	NA	13 015	NA	4688	3487	2829	2011	NA
Incidence rates per 1000 person-years	NA	28.0	NA	20.9	30.1	33.9	32.8	NA
Model 1	2724	1.11 (1.00-1.23)	.05	1 [Reference]	1.22 (0.93-1.62)	1.37 (1.02-1.84)	1.26 (0.90-1.77)	.08
Model 2	2632	1.09 (0.98-1.21)	.13	1 [Reference]	1.26 (0.95-1.68)	1.36 (1.00-1.84)	1.21 (0.85-1.71)	.16
Model 3	2598	1.08 (0.97-1.21)	.17	1 [Reference]	1.25 (0.93-1.67)	1.34 (0.98-1.84)	1.21 (0.84-1.74)	.18
**Impairment in attention and processing speed (Trail Making Test A)**								
Events, No.	NA	313	NA	71	75	91	76	NA
Person-years	NA	13 747	NA	5121	3761	2989	1876	NA
Incidence rates per 1000 person-years	NA	22.8	NA	13.9	19.9	30.4	40.5	NA
Model 1	2865	1.26 (1.12-1.42)	<.001^c^	1 [Reference]	0.97 (0.70-1.35)	1.31 (0.95-1.81)	1.74 (1.23-2.46)	<.001^c^
Model 2	2764	1.23 (1.09-1.40)	.001^c^	1 [Reference]	1.00 (0.71-1.40)	1.21 (0.87-1.70)	1.67 (1.16-2.39)	.005^c^
Model 3	2724	1.21 (1.05-1.38)	.006^c^	1 [Reference]	0.95 (0.67-1.34)	1.18 (0.83-1.67)	1.54 (1.05-2.27)	.03
**Impairment in executive function (Trail Making Test B)**								
Events, No.	NA	482	NA	138	140	127	77	NA
Person-years	NA	12 038	NA	4682	3315	2548	1493	NA
Incidence rates per 1000 person-years	NA	40.0	NA	29.5	42.2	49.8	51.6	NA
Model 1	2590	1.08 (0.98-1.19)	.13	1 [Reference]	0.94 (0.74-1.19)	1.19 (0.93-1.52)	1.15 (0.86-1.54)	.17
Model 2	2506	1.06 (0.96-1.17)	.27	1 [Reference]	0.90 (0.70-1.16)	1.12 (0.86-1.45)	1.09 (0.80-1.47)	.37
Model 3	2469	1.03 (0.93-1.15)	.57	1 [Reference]	0.90 (0.70-1.16)	1.13 (0.87-1.48)	0.99 (0.71-1.36)	.68

Abbreviations: eGFR, estimated glomerular filtration rate; HR, hazard ratio; NA, not applicable.

^a^ Per 1-SD increase.

^b^ Model 1 adjusted for age, sex, race and ethnicity, clinical center, educational level, and baseline cognitive score. Model 2 adjusted for all covariates in model 1 plus smoking, depression, alcohol use, physical activity, and body mass index. Model 3 adjusted for all covariates in model 2 plus systolic blood pressure, renin angiotensin aldosterone inhibitor use, baseline diabetes, baseline cardiovascular disease, white blood cell counts, and hemoglobin.

^c^ Significant after false discovery rate correction for testing multiple cognitive end points.

### Prospective Associations Between UPCR and Cognitive Impairment

A higher baseline UPCR was significantly associated with increased incidence of cognitive impairment across multiple domains (Table 3). In our fully adjusted model, each 1 SD higher log UPCR was associated with 21% increased risk of impairments in attention and processing speed (HR, 1.21; 95% CI, 1.05-1.41; *P* = .01) and 16% increased risk of impairment in executive function (HR, 1.16; 95% CI, 1.02-1.31; *P* = .02), which remained significant after FDR correction. When investigating the UPCR as an ordinal variable (Table 3), a nominally significant dose-response association between increasing UPCR and risk of global cognitive impairment was observed (HR, 1.26; 95% CI, 0.99-1.59; *P* = .04 for linear trend). In spline analyses of the log-transformed UPCR (eFigure 2 in Supplement 1), there was no evidence of nonlinear associations between UPCR and any of the cognition tests, with significant and nominally significant linear associations observed for tests of attention and processing speed and executive function. Consistent with the primary analyses of cognitive impairment, latent process mixed models identified significant associations of increasing proteinuria with longitudinal declines in global cognition, attention and processing speed, and executive function (eTable 4 in Supplement 1). In models further adjusting for the eGFR (eTable 3 in Supplement 1), associations of the continuous log UPCR with impairments in attention and processing speed (HR, 1.19; 95% CI, 1.02-1.40; *P* = .03) and executive function (HR, 1.15; 95% CI, 1.01-1.32; *P* = .04) remained nominally significant, with effect sizes consistent with those presented in model 3 (Table 2).

**Table 3.** Associations Between Baseline UPCR Stage and Incident Cognitive Impairment

Characteristic		No. of patients	Log UPCR		HR (95% CI) for UPCR category, mg/g			*P* value
			HR (95% CI)^a^	*P* value	<150	150-500	>500	
**Impairment in global cognition (Modified Mini-Mental State Examination)**								
Events, No.	NA		531	NA	251	121	159	NA
Person-years	NA		30 405	NA	17 557	5849	6999	NA
Incidence rates per 1000 person-years	NA		17.5	NA	14.3	20.7	22.7	NA
Model 1^b^	4081		1.18 (1.08-1.29)	<.001^c^	1 [Reference]	1.31 (1.05-1.63)	1.45 (1.17-1.78)	<.001^c^
Model 2^b^	3943		1.19 (1.08-1.30)	<.001^c^	1 [Reference]	1.29 (1.03-1.61)	1.49 (1.20-1.84)	<.001^c^
Model 3^b^	3920		1.09 (0.98-1.20)	.10	1 [Reference]	1.19 (0.95-1.50)	1.26 (0.99-1.59)	.04
**Impairment in verbal memory and delayed recall (Buschke Selective Reminding Test)**								
Events, No.	NA		333	NA	156	86	91	NA
Person-years	NA		11 731	NA	6892	2344	2495	NA
Incidence rates per 1000 person-years	NA		28.4	NA	22.6	36.7	36.5	NA
Model 1	2455		1.16 (1.03-1.31)	.02^c^	1 [Reference]	1.28 (0.97-1.68)	1.36 (1.03-1.79)	.02^c^
Model 2	2366		1.16 (1.02-1.32)	.02^c^	1 [Reference]	1.28 (0.97-1.68)	1.35 (1.02-1.79)	.03^c^
Model 3	2352		1.13 (0.99-1.29)	.08	1 [Reference]	1.24 (0.94-1.64)	1.26 (0.94-1.70)	.10
**Impairment in attention and processing speed (Trail Making Test A)**								
Events, No.	NA		288	NA	136	71	81	NA
Person-years	NA		12 413	NA	7432	2460	2521	NA
Incidence rates per 1000 person-years	NA		23.2	NA	18.3	28.9	32.1	NA
Model 1	2596		1.22 (1.07-1.40)	.003^c^	1 [Reference]	1.32 (0.99-1.77)	1.42 (1.05-1.91)	.01^c^
Model 2	2503		1.24 (1.08-1.42)	.002^c^	1 [Reference]	1.37 (1.01-1.85)	1.43 (1.06-1.94)	.01^c^
Model 3	2486		1.21 (1.05-1.41)	.01^c^	1 [Reference]	1.39 (1.02-1.89)	1.33 (0.96-1.84)	.05
**Impairment in executive function (Trail Making Test B)**								
Events, No.	NA		435	NA	225	106	104	NA
Person-years	NA		10 856	NA	6691	2079	2086	NA
Incidence rates per 1000 person-years	NA		40.1	NA	33.6	51.0	49.9	NA
Model 1	2346		1.19 (1.06-1.32)	.002^c^	1 [Reference]	1.36 (1.07-1.72)	1.42 (1.11-1.81)	.007^c^
Model 2	2267		1.19 (1.06-1.33)	.002^c^	1 [Reference]	1.36 (1.06-1.73)	1.32 (1.02-1.70)	.02^c^
Model 3	2252		1.16 (1.02-1.31)	.02^c^	1 [Reference]	1.31 (1.02-1.68)	1.19 (0.90-1.57)	.12

Abbreviations: HR, hazard ratio; NA, not applicable; UPCR, urinary protein to creatinine ratio.

^a^ Per 1-SD increase.

^b^ Model 1 adjusted for age, sex, race and ethnicity, clinical center, educational level, and baseline cognitive score. Model 2 adjusted for all covariables in model 1 plus smoking, depression, alcohol use, physical activity, and body mass index. Model 3 adjusted for all covariables in model 2 plus systolic blood pressure, renin angiotensin aldosterone inhibitor use, baseline diabetes, baseline cardiovascular disease, white blood cell counts, and hemoglobin.

^c^ Significant after false discovery rate correction for testing multiple cognitive end points.

### Joint Prospective Associations of eGFR and UPCR With Cognitive Impairment

**Figure 2.**
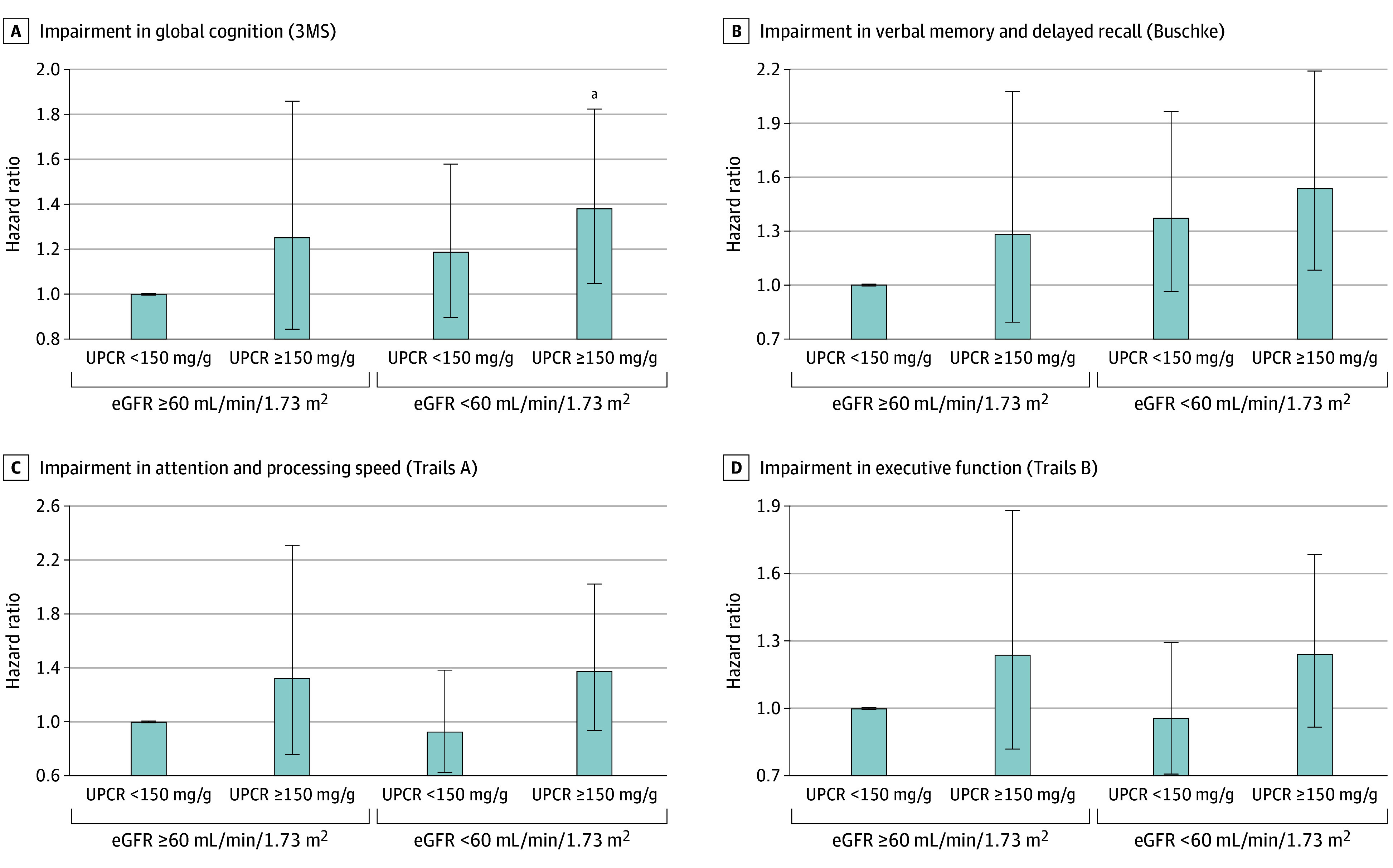
Multivariable-Adjusted Hazard Ratios Depicting the Joint Associations of Estimated Glomerular Filtration Rate (eGFR) and Urine Protein to Creatinine Ratio (UPCR) With the Risk of Cognitive Impairment A, Impairment in global cognition, assessed by the Modified Mini-Mental State Examination (3MS). B, Impairment in verbal memory and delayed recall, assessed by the Buschke Selective Reminding Test (Buschke). C, Impairment in attention and processing speed, assessed by the Trail Making Test A (Trails A). D, Impairment in executive function, assessed by the Trail Making Test B (Trails B). ^a^Significant after false discovery rate correction for testing multiple cognitive end points.

Increased incident impairment in global cognition and verbal memory and delayed recall was associated with a combined higher UPCR and lower eGFR (Figure 2; eTable 5 in Supplement 1). In our fully adjusted model, the most advanced eGFR and UPCR stages were associated with significant 38% increased risk of impairment in global cognition (HR, 1.38; 95% CI, 1.05-1.82; *P* = .003) and nominally significant 54% increased risk of impairment in verbal memory and recall (HR, 1.54; 95% CI, 1.08-2.19; *P* = .02). The eGFR and UPCR appeared complementary, with no evidence of their interactions on cognition end points (eTable 3 in Supplement 1). There were no associations of combined eGFR and UPCR with impairments in attention and processing speed or executive function.

### Subgroup and Sensitivity Analyses

There was no heterogeneity of associations observed across strata of age, sex, race and ethnicity, and diabetes status after adjusting for the number of subgroups tested (eFigure 3 and eFigure 4 in Supplement 1). Likewise, sensitivity analyses yielded similar results when using the race-neutral creatinine and cystatin C CRIC eGFR equation (eTable 6 in Supplement 1), substituting UACR for UPCR (eTable 7 in Supplement 1), using alternate definitions of cognitive impairment (eTable 8 and eTable 9 in Supplement 1), applying 4 or 5 knots in spline analyses (eTable 10 in Supplement 1), and accounting for competing risks of death (eTables 11 and 12 in Supplement 1).

## Discussion

In this cohort study of a large and diverse sample of patients with CKD, more advanced CKD stage was prospectively associated with increased incidence of cognitive impairment independent of known risk factors. Specifically, more advanced proteinuria was significantly associated with impairments in attention and processing speed as well as executive function. Although a decreased eGFR was also associated with attention and processing speed, proteinuria largely explained this association. Joint analyses revealed that the most advanced combined eGFR and UPCR stage was associated with impairments in global cognition, but no interactions between these kidney function measures were observed. There was also no evidence to support nonlinear associations between kidney function measures and cognitive impairment. In total, our results suggested that increased risk of cognitive impairment was associated with more advanced CKD stage, providing important clues for risk stratification in this high-risk population.

Research to better understand the pathophysiological mechanisms linking CKD with cognitive dysfunction is ongoing, and several credible hypotheses have been proposed. For example, scientists have pointed out the anatomical and functional similarity of the microvasculature in the kidney and brain.^12,29^ With both organs susceptible to microvascular injury caused by risk factors including hypertension and diabetes, CKD and cognitive impairment may share underlying mechanisms.^30,31,32,33^ Because CKD is known to exacerbate hypertension,^34^ the hemodynamic consequences of CKD progression could explain the increased risk of cognitive impairment experienced in this patient population. Supporting this finding, evidence suggests that vascular associations with dementia may be more prominent among patients with CKD compared with the general population.^35^ We consistently observed some attenuation in associations between CKD and cognitive impairment after adjustment for clinical risk factors, including hypertension. Still, clinical risk factors did not entirely explain the associations, suggesting that other mechanisms may be associated with cognitive decline in CKD. Scientists have speculated that uremic toxins, or metabolites that accumulate in patients with CKD due to decreased kidney filtration and tubular secretion, could play a role in the increased risk of cognitive impairment.^36^ Metabolites such as kynurenine and indoxyl sulfate can negatively alter endothelial cells that comprise the blood-brain barrier.^37,38,39,40,41^ These alterations might promote vascular injury^42,43^ and increase blood-brain barrier permeability,^44,45^ enabling passage of neurotoxic molecules from the periphery to brain tissue.^46^ Other CKD-related conditions, such as abnormal bone mineral metabolism,^46,47,48^ chronic inflammation and oxidative stress,^21,49^ sleep disorders,^50,51^ and anemia,^52^ may also contribute to the observed associations between kidney and brain health as shown in prior studies.

The UPCR was prospectively associated with impairments in attention and processing speed as well as executive function, maintaining nominal significance after adjustment for the eGFR. In contrast, the eGFR’s association with impairment in attention and processing speed was largely attenuated after UPCR adjustment. Only a few community-based studies^5,10,11^ have previously assessed both the eGFR and proteinuria in association with cognition. All reported persistent associations of albuminuria with cognitive impairment end points after adjusting for the eGFR, while associations of the eGFR with cognitive impairment end points after adjusting for the UACR were either inconsistent or null.^5,11^ For example, the Atherosclerosis Risk in Communities (ARIC) study found that each IQR increase in the UACR was associated with a significant 1.15-fold increased risk of incident dementia among those aged 54 to 74 years and a 1.27-fold increased risk of incident dementia among those aged 70 to 90 years in models including the eGFR.^5^ In contrast, using the creatinine-cystatin C eGFR, associations with dementia were no longer significant among those aged 54 to 74 years after UACR adjustment.^10^ In total, we extend prior work by showing that the UPCR may be a more robust factor associated with future cognitive impairment than the eGFR in an exclusive population of patients with CKD across distinct cognitive domains.

Joint analyses of the eGFR and the UPCR suggested complementary associations with global cognitive impairment, with the highest risks in the most severe eGFR and UPCR category. Similarly, the highest risk of dementia among ARIC study participants was observed in the most severe albuminuria and eGFR grouping.^5^ Likewise, Takae and colleagues^11^ showed graded increases in relative risks of Alzheimer disease and vascular dementias with joint worsening of the UACR and the eGFR. Despite findings from individual analyses that the UPCR may be a more robust determinant than the eGFR, their complementary nature suggests the potential relevance of both measures for risk stratification purposes.

### Strengths and Limitations

This study has some strengths. The CRIC Study provides longitudinal measurements on a battery of cognitive tests, allowing for the assessment of global cognition and several relevant cognitive domains. Furthermore, the CRIC Study includes patients with CKD spanning a wide spectrum of disease severity and uses rigorous measures of the eGFR and urinary protein. In addition, our study integrates both creatinine and cystatin C equations for eGFR estimation, which is particularly relevant for older adults whose serum creatinine level may be influenced by low muscle mass.

However, our study also has limitations. Urinary protein was evaluated rather than urinary albumin due to the availability of the latter measure in only a small subsample of CRIC participants. Although both urinary protein and urinary albumin are associated with CKD sequelae,^53,54^ UPCR thresholds have not been rigorously associated with CKD staging guidelines. For the present study, we used UPCR thresholds of 150 and 500 mg/g based on the approximate correlations of these values with standard UACR values of 30 and 300 mg/g.^16^ Sensitivity analyses examining the association of the UACR with global cognition in the subsample with measured UACR values were similar to analyses using the UPCR, suggesting that our findings were robust. In addition, the CRIC Study excluded individuals with end-stage kidney disease, which might limit the generalizability of our findings to those in the most advanced stage of CKD. With respect to the annual or biennial cognitive tests used, practice effects and ceiling effects may have reduced cognitive change over time,^55^ potentially attenuating observed associations. Furthermore, we cannot rule out differential attrition among those with cognitive impairment, who may have been more likely to be lost to follow-up compared with those with normal cognition. Given that decreased kidney function was generally associated with increased incidence of cognitive impairment, it is likely that such bias would attenuate our findings. Furthermore, while we adjusted for a large number of lifestyle and clinical risk factors, residual confounding may persist.

## Conclusions

This cohort study found that more advanced CKD stage was prospectively associated with increased incidence of cognitive impairment. Although significant findings were observed in individual analyses of both the eGFR and the UPCR, the UPCR was shown to be a more robust determinant when modeled together. Joint analyses revealed complementary associations but no interactions between the eGFR and the UPCR, suggesting the value of both measures for risk stratification purposes. These findings underscore CKD severity as a risk factor for cognitive decline across the CKD spectrum.

## Notes

Supplement 1. **eTable 1.** Baseline Characteristics of Participants Included in the Analytic Sample for Each Cognitive Test **eTable 2.** Associations of CKD Based eGFR With Longitudinal Changes in Cognitive Tests in Fully Adjusted Models (Model 3) That Using Latent Mixed Model With Beta Link Function **eTable 3.** Associations of CKD Based eGFR and Proteinuria With Cognitive Impairment in Fully Adjusted Models (Model 3) That Also Includes Both Kidney Function Variables **eTable 4.** Associations of Proteinuria With Longitudinal Changes in Cognitive Tests in Fully Adjusted Models (Model 3) That Using Latent Mixed Model With Beta Link Function **eTable 5.** Joint Prospective Associations Between eGFR-Based CKD and Proteinuria With Cognitive Impairment **eTable 6.** Associations of CKD Based eGFR With Cognitive Impairment In Fully Adjusted Models (Model 3) Comparing eGFR as Estimated by the Race-Neutral, Creatinine and Cystatin C-Based CKD-Epi and CRIC Equations **eTable 7.** Associations of Proteinuria Compared to Albuminuria With Cognitive Impairment in Fully Adjusted Models **eTable 8.** Associations of CKD Based eGFR With Cognitive Impairment in Fully Adjusted Models (Model 3) That Defined Cognitive Impairment Based on a 5% Worsening of Cognition From Baseline **eTable 9.** Associations of Proteinuria With Cognitive Impairment in Fully Adjusted Models (Model 3) That Defined Cognitive Impairment as a 5% Worsening Since Baseline **eTable 10.** Restrict Cubic Spline of CKD Based eGFR and Proteinuria With Cognitive Impairment in Fully Adjusted Models (Model 3) That Used Different Knots **eTable 11.** Associations of CKD Based eGFR With Cognitive Impairment in Fully Adjusted Models (Model 3) That With Death as Competing Risk Event **eTable 12.** Associations of Proteinuria With Cognitive Impairment in Fully Adjusted Models (Model 3) That With Death as Competing Risk Event **eFigure 1.** Restricted Cubic Spline Plots of Estimated Glomerular Filtration Rate **eFigure 2.** Restricted Cubic Spline Plots of Log-Transformed Urinary Protein-to-Creatinine Ratio **eFigure 3.** Multivariable Adjusted Hazard Ratios for Cognitive Impairment Corresponding to Each Standard Deviation Decrease in eGFR **eFigure 4.** Multivariable Adjusted Hazard Ratios for Cognitive Impairment Corresponding to Each Standard Deviation Increase in UPCR
eMethods.

eReferences.

Supplement 2. **Nonauthor Collaborators.** Chronic Renal Insufficiency Cohort (CRIC) Study Investigators

Supplement 3.
Data Sharing Statement

## References

[1] Nichols E, Steinmetz JD, Vollset SE, ; GBD 2019 Dementia Forecasting Collaborators. Estimation of the global prevalence of dementia in 2019 and forecasted prevalence in 2050: an analysis for the Global Burden of Disease Study 2019. Lancet Public Health. 2022;7(2):e105-e125. doi:10.1016/S2468-2667(21)00249-8 34998485 PMC8810394

[2] Ikram MA. Chronic kidney disease and dementia: an epidemiological perspective. Nat Rev Nephrol. 2025;21(8):525-535. doi:10.1038/s41581-025-00967-w 40404981

[3] Seliger SL, Siscovick DS, Stehman-Breen CO, . Moderate renal impairment and risk of dementia among older adults: the Cardiovascular Health Cognition Study. J Am Soc Nephrol. 2004;15(7):1904-1911. doi:10.1097/01.ASN.0000131529.60019.FA 15213280

[4] Xu H, Garcia-Ptacek S, Trevisan M, . Kidney function, kidney function decline, and the risk of dementia in older adults: a registry-based study. Neurology. 2021;96(24):e2956-e2965. doi:10.1212/WNL.0000000000012113 33952656 PMC8253567

[5] Scheppach JB, Coresh J, Wu A, . Albuminuria and estimated GFR as risk factors for dementia in midlife and older age: findings from the ARIC study. Am J Kidney Dis. 2020;76(6):775-783. doi:10.1053/j.ajkd.2020.03.015 32428540 PMC7669634

[6] Buchman AS, Tanne D, Boyle PA, Shah RC, Leurgans SE, Bennett DA. Kidney function is associated with the rate of cognitive decline in the elderly. Neurology. 2009;73(12):920-927. doi:10.1212/WNL.0b013e3181b72629 19657107 PMC2754333

[7] Slinin Y, Paudel ML, Ishani A, ; Osteoporotic Fractures in Men Study Group. Kidney function and cognitive performance and decline in older men. J Am Geriatr Soc. 2008;56(11):2082-2088. doi:10.1111/j.1532-5415.2008.01936.x 18795984 PMC3108463

[8] Yaffe K, Kurella-Tamura M, Ackerson L, ; CRIC Study Investigators. Higher levels of cystatin C are associated with worse cognitive function in older adults with chronic kidney disease: the Chronic Renal Insufficiency Cohort Cognitive Study. J Am Geriatr Soc. 2014;62(9):1623-1629. doi:10.1111/jgs.12986 25125225 PMC4201363

[9] Yaffe K, Ackerson L, Kurella Tamura M, ; Chronic Renal Insufficiency Cohort Investigators. Chronic kidney disease and cognitive function in older adults: findings from the Chronic Renal Insufficiency Cohort Cognitive Study. J Am Geriatr Soc. 2010;58(2):338-345. doi:10.1111/j.1532-5415.2009.02670.x 20374407 PMC2852884

[10] Kurella Tamura M, Muntner P, Wadley V, . Albuminuria, kidney function, and the incidence of cognitive impairment among adults in the United States. Am J Kidney Dis. 2011;58(5):756-763. doi:10.1053/j.ajkd.2011.05.027 21816528 PMC3199339

[11] Takae K, Hata J, Ohara T, . Albuminuria increases the risks for both Alzheimer disease and vascular dementia in community-dwelling Japanese elderly: the Hisayama Study. J Am Heart Assoc. 2018;7(2):e006693. doi:10.1161/JAHA.117.006693 29353232 PMC5850144

[12] Knopman DS. Invited commentary: albuminuria and microvascular disease of the brain—a shared pathophysiology. Am J Epidemiol. 2010;171(3):287-289. doi:10.1093/aje/kwp429 20061365 PMC2842197

[13] Lash JP, Go AS, Appel LJ, ; Chronic Renal Insufficiency Cohort (CRIC) Study Group. Chronic Renal Insufficiency Cohort (CRIC) Study: baseline characteristics and associations with kidney function. Clin J Am Soc Nephrol. 2009;4(8):1302-1311. doi:10.2215/CJN.00070109 19541818 PMC2723966

[14] Inker LA, Eneanya ND, Coresh J, ; Chronic Kidney Disease Epidemiology Collaboration. New creatinine- and cystatin C–based equations to estimate GFR without race. N Engl J Med. 2021;385(19):1737-1749. doi:10.1056/NEJMoa2102953 34554658 PMC8822996

[15] 2024 Annual data report. US Renal Data System. Accessed June 2, 2025. https://usrds-adr.niddk.nih.gov/2024

[16] Kidney Disease: Improving Global Outcomes (KDIGO) CKD Work Group. KDIGO 2012 Clinical Practice Guideline for the Evaluation and Management of Chronic Kidney Disease. *Kidney International* supplements. 2013;3:1-150. Accessed January 7, 2026. https://kdigo.org/wp-content/uploads/2017/02/KDIGO_2012_CKD_GL.pdf 10.1016/j.kisu.2017.10.001 PMC6341011 30681074

[17] Stevens PE, Ahmed SB, Carrero JJ, ; Kidney Disease: Improving Global Outcomes (KDIGO) CKD Work Group. KDIGO 2024 Clinical Practice Guideline for the Evaluation and Management of Chronic Kidney Disease. Kidney Int. 2024;105(4S):S117-S314. doi:10.1016/j.kint.2023.10.018 38490803

[18] Hodel NC, Rentsch KM, Paris DH, Mayr M. Methods for diagnosing proteinuria—when to use which test and why: a review. Am J Kidney Dis. 2025;85(5):618-628. doi:10.1053/j.ajkd.2024.09.017 39706243

[19] Xiao C, Tamura MK, Pan Y, ; CRIC Study Investigators. Clonal hematopoiesis of indeterminate potential is associated with reduced risk of cognitive impairment in patients with chronic kidney disease. Alzheimers Dement. 2024;20(10):6960-6971. doi:10.1002/alz.14182 39115897 PMC11485087

[20] Babroudi S, Tighiouart H, Schrauben SJ, ; CRIC Study Investigators. Blood pressure, incident cognitive impairment, and severity of CKD: findings from the Chronic Renal Insufficiency Cohort (CRIC) Study. Am J Kidney Dis. 2023;82(4):443-453.e1. doi:10.1053/j.ajkd.2023.03.012 37245689 PMC10526961

[21] Kurella Tamura M, Tam K, Vittinghoff E, ; CRIC Study Investigators. Inflammatory markers and risk for cognitive decline in chronic kidney disease: the CRIC Study. Kidney Int Rep. 2017;2(2):192-200. doi:10.1016/j.ekir.2016.10.007 28439566 PMC5399682

[22] Lin FR, Yaffe K, Xia J, ; Health ABC Study Group. Hearing loss and cognitive decline in older adults. JAMA Intern Med. 2013;173(4):293-299. doi:10.1001/jamainternmed.2013.1868 23337978 PMC3869227

[23] Bernick C, Katz R, Smith NL, ; Cardiovascular Health Study Collaborative Research Group. Statins and cognitive function in the elderly: the Cardiovascular Health Study. Neurology. 2005;65(9):1388-1394. doi:10.1212/01.wnl.0000182897.18229.ec 16275825

[24] Lidgard B, Bansal N, Zelnick LR, ; CRIC Study Investigators. Association of proximal tubular secretory clearance with long-term decline in cognitive function. J Am Soc Nephrol. 2022;33(7):1391-1401. doi:10.1681/ASN.2021111435 35444055 PMC9257801

[25] Ganiayre J, Commenges D, Letenneur L. A latent process model for dementia and psychometric tests. Lifetime Data Anal. 2008;14(2):115-133. doi:10.1007/s10985-007-9057-x 17874295

[26] Proust C, Jacqmin-Gadda H, Taylor JMG, Ganiayre J, Commenges D. A nonlinear model with latent process for cognitive evolution using multivariate longitudinal data. Biometrics. 2006;62(4):1014-1024. doi:10.1111/j.1541-0420.2006.00573.x 17156275 PMC1930148

[27] Jacqmin-Gadda H, Proust-Lima C, Amiéva H. Semi-parametric latent process model for longitudinal ordinal data: application to cognitive decline. Stat Med. 2010;29(26):2723-2731. doi:10.1002/sim.4035 20809483

[28] Hsu CY, Yang W, Parikh RV, et al; CRIC Study Investigators. Race, genetic ancestry, and estimating kidney function in CKD. N Engl J Med. 2021;385(19):1750-1760. doi:10.1056/NEJMoa2103753 34554660 PMC8994696

[29] Ito S, Nagasawa T, Abe M, Mori T. Strain vessel hypothesis: a viewpoint for linkage of albuminuria and cerebro-cardiovascular risk. Hypertens Res. 2009;32(2):115-121. doi:10.1038/hr.2008.27 19262469

[30] Hainsworth AH, Markus HS, Schneider JA. Cerebral small vessel disease, hypertension, and vascular contributions to cognitive impairment and dementia. Hypertension. 2024;81(1):75-86. doi:10.1161/HYPERTENSIONAHA.123.19943 38044814 PMC10734789

[31] Saeed A, Lopez O, Cohen A, Reis SE. Cardiovascular disease and Alzheimer’s disease: the heart-brain axis. J Am Heart Assoc. 2023;12(21):e030780. doi:10.1161/JAHA.123.030780 37929715 PMC10727398

[32] Horton WB, Barrett EJ. Microvascular dysfunction in diabetes mellitus and cardiometabolic disease. Endocr Rev. 2021;42(1):29-55. doi:10.1210/endrev/bnaa025 33125468 PMC7846151

[33] Miglinas M, Cesniene U, Janusaite MM, Vinikovas A. Cerebrovascular disease and cognition in chronic kidney disease patients. Front Cardiovasc Med. 2020;7:96. doi:10.3389/fcvm.2020.00096 32582768 PMC7283453

[34] Burnier M, Damianaki A. Hypertension as cardiovascular risk factor in chronic kidney disease. Circ Res. 2023;132(8):1050-1063. doi:10.1161/CIRCRESAHA.122.321762 37053276

[35] Scheppach JB, Wu A, Gottesman RF, . Association of kidney function measures with signs of neurodegeneration and small vessel disease on brain magnetic resonance imaging: the Atherosclerosis Risk in Communities (ARIC) study. Am J Kidney Dis. 2023;81(3):261-269. doi:10.1053/j.ajkd.2022.07.013 36179945 PMC9974563

[36] Vanholder R, De Smet R, Glorieux G, ; European Uremic Toxin Work Group (EUTox). Review on uremic toxins: classification, concentration, and interindividual variability. Kidney Int. 2003;63(5):1934-1943. doi:10.1046/j.1523-1755.2003.00924.x 12675874

[37] Watson CP, Pazarentzos E, Fidanboylu M, Padilla B, Brown R, Thomas SA. The transporter and permeability interactions of asymmetric dimethylarginine (ADMA) and L-arginine with the human blood–brain barrier in vitro. Brain Res. 2016;1648(pt A):232-242. doi:10.1016/j.brainres.2016.07.026 27431938 PMC5042357

[38] Yilmaz MI, Sonmez A, Saglam M, . FGF-23 and vascular dysfunction in patients with stage 3 and 4 chronic kidney disease. Kidney Int. 2010;78(7):679-685. doi:10.1038/ki.2010.194 20613714

[39] Huang M, Wei R, Wang Y, Su T, Li P, Chen X. The uremic toxin hippurate promotes endothelial dysfunction via the activation of Drp1-mediated mitochondrial fission. Redox Biol. 2018;16:303-313. doi:10.1016/j.redox.2018.03.010 29573704 PMC5953222

[40] Hernandez L, Ward LJ, Arefin S, ; GOING-FWD Collaborators. Blood-brain barrier and gut barrier dysfunction in chronic kidney disease with a focus on circulating biomarkers and tight junction proteins. Sci Rep. 2022;12(1):4414. doi:10.1038/s41598-022-08387-7 35292710 PMC8924178

[41] Fang C, Lau WL, Sun J, . Chronic kidney disease promotes cerebral microhemorrhage formation. J Neuroinflammation. 2023;20(1):51. doi:10.1186/s12974-023-02703-2 36841828 PMC9960195

[42] Wardlaw JM, Smith C, Dichgans M. Small vessel disease: mechanisms and clinical implications. Lancet Neurol. 2019;18(7):684-696. doi:10.1016/S1474-4422(19)30079-1 31097385

[43] Quick S, Moss J, Rajani RM, Williams A. A vessel for change: endothelial dysfunction in cerebral small vessel disease. Trends Neurosci. 2021;44(4):289-305. doi:10.1016/j.tins.2020.11.003 33308877

[44] Mazumder MK, Paul R, Bhattacharya P, Borah A. neurological sequel of chronic kidney disease: from diminished acetylcholinesterase activity to mitochondrial dysfunctions, oxidative stress and inflammation in mice brain. Sci Rep. 2019;9(1):3097. doi:10.1038/s41598-018-37935-3 30816118 PMC6395638

[45] Viggiano D, Wagner CA, Martino G, . Mechanisms of cognitive dysfunction in CKD. Nat Rev Nephrol. 2020;16(8):452-469. doi:10.1038/s41581-020-0266-9 32235904

[46] McGrath ER, Himali JJ, Levy D, . Circulating fibroblast growth factor 23 levels and incident dementia: the Framingham Heart Study. PLoS One. 2019;14(3):e0213321. doi:10.1371/journal.pone.0213321 30830941 PMC6398923

[47] Zou D, Wu W, He Y, Ma S, Gao J. The role of klotho in chronic kidney disease. BMC Nephrol. 2018;19(1):285. doi:10.1186/s12882-018-1094-z 30348110 PMC6198535

[48] Hagström E, Kilander L, Nylander R, . Plasma parathyroid hormone is associated with vascular dementia and cerebral hyperintensities in two community-based cohorts. J Clin Endocrinol Metab. 2014;99(11):4181-4189. doi:10.1210/jc.2014-1736 25140397

[49] Peyster E, Chen J, Feldman HI, ; CRIC Study Investigators. Inflammation and arterial stiffness in chronic kidney disease: findings from the CRIC Study. Am J Hypertens. 2017;30(4):400-408. doi:10.1093/ajh/hpw164 28391349 PMC5861572

[50] Lyons OD. Sleep disorders in chronic kidney disease. Nat Rev Nephrol. 2024;20(10):690-700. doi:10.1038/s41581-024-00848-8 38789686

[51] Wennberg AMV, Wu MN, Rosenberg PB, Spira AP. Sleep disturbance, cognitive decline, and dementia: a review. Semin Neurol. 2017;37(4):395-406. doi:10.1055/s-0037-1604351 28837986 PMC5910033

[52] Arnold R, Issar T, Krishnan AV, Pussell BA. Neurological complications in chronic kidney disease. JRSM Cardiovasc Dis. Published online November 3, 2016. doi:10.1177/2048004016677687 27867500 PMC5102165

[53] Fisher H, Hsu CY, Vittinghoff E, Lin F, Bansal N. Comparison of associations of urine protein-creatinine ratio versus albumin-creatinine ratio with complications of CKD: a cross-sectional analysis. Am J Kidney Dis. 2013;62(6):1102-1108. doi:10.1053/j.ajkd.2013.07.013 24041612 PMC3840083

[54] Heerspink HJL, Chadban SJ. Comparison of urine albumin-to-creatinine ratio (UACR) and protein-to-creatinine ratio (UPCR) as markers of kidney and cardiovascular disease risk: a multinational, patient-level meta-analysis. J Am Soc Nephrol. 2025;36(10S):10.1681/ASN.20259y5kcnm9. doi:10.1681/ASN.20259y5kcnm9

[55] Proust-Lima C, Dartigues JF, Jacqmin-Gadda H. Misuse of the linear mixed model when evaluating risk factors of cognitive decline. Am J Epidemiol. 2011;174(9):1077-1088. doi:10.1093/aje/kwr243 21965187 PMC3551607

