# Supplementary material for: Chronic Kidney Disease Severity and Risk of Cognitive Impairment
Source: JAMA Netw Open. 2026 Feb 17;9(2):e2559834. doi: 10.1001/jamanetworkopen.2025.59834 (PMC12914485; doi:10.1001/jamanetworkopen.2025.59834)
Supplement: Supplement 1. — eTable 1. Baseline Characteristics of Participants Included in the Analytic Sample for Each Cognitive Test eTable 2. Associations of CKD Based eGFR With Longitudinal Changes in Cognitive Tests in Fully Adjusted Models (Model 3) That Using Latent Mixed Model With Beta Link Function eTable 3. Associations of CKD Based eGFR and Proteinuria With Cognitive Impairment in Fully Adjusted Models (Model 3) That Also Includes Both Kidney Function Variables eTable 4. Associations of Proteinuria With Longitudinal Changes in Cognitive Tests in Fully Adjusted Models (Model 3) That Using Latent Mixed Model With Beta Link Function eTable 5. Joint Prospective Associations Between eGFR-Based CKD and Proteinuria With Cognitive Impairment eTable 6. Associations of CKD Based eGFR With Cognitive Impairment In Fully Adjusted Models (Model 3) Comparing eGFR as Estimated by the Race-Neutral, Creatinine and Cystatin C-Based CKD-Epi and CRIC Equations eTable 7. Associations of Proteinuria Compared to Albuminuria With Cognitive Impairment in Fully Adjusted Models eTable 8. Associations of CKD Based eGFR With Cognitive Impairment in Fully Adjusted Models (Model 3) That Defined Cognitive Impairment Based on a 5% Worsening of Cognition From Baseline eTable 9. Associations of Proteinuria With Cognitive Impairment in Fully Adjusted Models (Model 3) That Defined Cognitive Impairment as a 5% Worsening Since Baseline eTable 10. Restrict Cubic Spline of CKD Based eGFR and Proteinuria With Cognitive Impairment in Fully Adjusted Models (Model 3) That Used Different Knots eTable 11. Associations of CKD Based eGFR With Cognitive Impairment in Fully Adjusted Models (Model 3) That With Death as Competing Risk Event eTable 12. Associations of Proteinuria With Cognitive Impairment in Fully Adjusted Models (Model 3) That With Death as Competing Risk Event eFigure 1. Restricted Cubic Spline Plots of Estimated Glomerular Filtration Rate eFigure 2. Restricted Cubic Spline Plots of Log-Transformed Urinary Protein-to [file jamanetwopen-e2559834-s001.pdf]

Supplementary Online Content

Huang Z, Yaffe K, Li C, et al; Chronic Renal Insufficiency Cohort (CRIC) Study Investigators. Chronic kidney disease severity and risk of cognitive impairment. *JAMA Netw Open.* 2026;9(2):e2559834. doi:10.1001/jamanetworkopen.2025.59834

eTable 1. Baseline Characteristics of Participants Included in the Analytic Sample for Each Cognitive Test
eTable 2. Associations of CKD Based eGFR With Longitudinal Changes in Cognitive Tests in Fully Adjusted Models (Model 3) That Using Latent Mixed Model With Beta Link Function
eTable 3. Associations of CKD Based eGFR and Proteinuria With Cognitive Impairment in Fully Adjusted Models (Model 3) That Also Includes Both Kidney Function Variables
eTable 4. Associations of Proteinuria With Longitudinal Changes in Cognitive Tests in Fully Adjusted Models (Model 3) That Using Latent Mixed Model With Beta Link Function
eTable 5. Joint Prospective Associations Between eGFR-Based CKD and Proteinuria With Cognitive Impairment
eTable 6. Associations of CKD Based eGFR With Cognitive Impairment In Fully Adjusted Models (Model 3) Comparing eGFR as Estimated by the Race-Neutral, Creatinine and Cystatin C-Based CKD-Epi and CRIC Equations
eTable 7. Associations of Proteinuria Compared to Albuminuria With Cognitive Impairment in Fully Adjusted Models
eTable 8. Associations of CKD Based eGFR With Cognitive Impairment in Fully Adjusted Models (Model 3) That Defined Cognitive Impairment Based on a 5% Worsening of Cognition From Baseline
eTable 9. Associations of Proteinuria With Cognitive Impairment in Fully Adjusted Models (Model 3) That Defined Cognitive Impairment as a 5% Worsening Since Baseline
eTable 10. Restrict Cubic Spline of CKD Based eGFR and Proteinuria With Cognitive Impairment in Fully Adjusted Models (Model 3) That Used Different Knots
eTable 11. Associations of CKD Based eGFR With Cognitive Impairment in Fully Adjusted Models (Model 3) That With Death as Competing Risk Event
eTable 12. Associations of Proteinuria With Cognitive Impairment in Fully Adjusted Models (Model 3) That With Death as Competing Risk Event
eFigure 1. Restricted Cubic Spline Plots of Estimated Glomerular Filtration Rate
eFigure 2. Restricted Cubic Spline Plots of Log-Transformed Urinary Protein-to-Creatinine Ratio
eFigure 3. Multivariable Adjusted Hazard Ratios for Cognitive Impairment Corresponding to Each Standard Deviation Decrease in eGFR
eFigure 4. Multivariable Adjusted Hazard Ratios for Cognitive Impairment Corresponding to Each Standard Deviation Increase in UPCR
eMethods.
eReferences.

This supplementary material has been provided by the authors to give readers additional information about their work.

© 2026 Huang Z et al. *JAMA Network Open.*

eTable 1. Baseline Characteristics of Participants Included in the Analytic Sample for Each Cognitive Test

Characteristic	3MS (n=4259)	Buschke (n=2724)	Trails A (n=2865)	Trails B (n=2590)
Age, years, mean (SD)	59.4 (10.6)	63.2 (9.6)	63.5 (9.7)	63.1 (9.8)
Male, n (%)	2395 (56.2)	1461 (53.6)	1622 (56.6)	1456 (56.2)
Race/ethnicity, n (%)				
Non-Hispanic White	2009 (47.2)	1299 (47.7)	1413 (49.3)	1366 (52.7)
Non-Hispanic Black	1756 (41.2)	1057 (38.8)	1106 (38.6)	930 (35.9)
Hispanic	343 (8.1)	258 (9.5)	230 (8.0)	181 (7.0)
Other	151 (3.5)	110 (4.0)	116 (4.0)	113 (4.4)
Education, n (%)				
Less than high school	533 (12.5)	387 (14.2)	382 (13.3)	255 (9.8)
High school	764 (17.9)	470 (17.3)	483 (16.9)	429 (16.6)
Some college	1339 (31.4)	817 (30.0)	868 (30.3)	812 (31.4)
College degree and higher	1623 (38.1)	1050 (38.5)	1132 (39.5)	1094 (42.2)
Depression, n (%)	586 (14.2)	368 (13.8)	381 (13.5)	339 (13.3)
Current Smoker, n (%)	485 (11.4)	229 (8.4)	253 (8.8)	223 (8.6)
Alcohol use, n (%)	2825 (66.3)	1595 (58.8)	1707 (59.8)	1605 (62.2)
Physical activity, METs/wk, mean (SD)	206.3 (144.6)	192.5 (132.5)	190.7 (130.8)	195.5 (131.3)
BMI, kg/m^2, mean (SD)	32.4 (7.6)	32.1 (7.6)	32.0 (7.5)	32.0 (7.6)
Systolic BP, mm Hg, mean (SD)	126.6 (20.1)	126.2 (20.2)	125.5 (19.7)	124.7 (19.1)
ACE inhibitors or ARBs use, n (%)	2911 (68.8)	1821 (67.1)	1921 (67.4)	1740 (67.6)
Hypertension, n (%)	3628 (85.2)	2464 (90.5)	2590 (90.5)	2321 (89.7)
Diabetes mellitus, n (%)	2037 (47.8)	1256 (46.1)	1292 (45.1)	1132 (43.7)
Self-reported cardiovascular disease, n (%)	1334 (31.3)	998 (36.6)	1034 (36.1)	906 (35.0)
White blood cell count, per µL, median (IQR)	6.3 (5.1, 7.7)	6.2 (5.1, 7.5)	6.2 (5.1, 7.5)	6.2 (5.1, 7.5)
Hemoglobin, g/dL, mean (SD)	12.8 (1.7)	12.8 (1.7)	12.9 (1.7)	12.9 (1.7)
eGFR, ml/min per 1.73 m^2, mean (SD)	53.7 (19.3)	51.6 (22.7)	52.4 (22.3)	53.3 (22.1)
eGFR category, ml/min per 1.73 m^2, n (%)				
G2 (≥60)	1499 (35.2)	957 (35.1)	1038 (36.2)	973 (37.6)
G3a (45-59)	1210 (28.4)	700 (25.7)	738 (25.8)	675 (26.1)
G3b (30-44)	1101 (25.9)	589 (21.6)	629 (22.0)	564 (21.8)
G4&G5 (<30)	449 (10.5)	478 (17.5)	460 (16.1)	378 (14.6)
UPCR, mg/g, median (IQR)	130 (50, 550)	130 (60, 490)	130 (60, 460)	120 (60, 430)
UPCR category, mg/g, n (%)				
No to mild proteinuria (<150)	2168 (53.3)	1273 (53.4)	1371 (54.4)	1270 (55.7)
Moderate proteinuria (150-500)	838 (20.6)	526 (22.1)	556 (22.0)	495 (21.7)
Severe proteinuria (>500)	1061 (26.1)	586 (24.6)	595 (23.6)	514 (22.6)

© 2026 Huang Z et al. *JAMA Network Open.*

Abbreviations: BMI, body mass index; BP, blood pressure; ACE, angiotensin-converting enzyme; ARB, angiotensin receptor blocker; eGFR, estimated glomerular filtration rate; UPCR, urine protein-creatinine ratio

© 2026 Huang Z et al. *JAMA Network Open.*

eTable 2. Associations of CKD Based eGFR With Longitudinal Changes in Cognitive Tests in Fully Adjusted Models (Model 3) That Using Latent Mixed Model With Beta Link Function[a]

Variable	No. of subject	No. of observation	Continuous eGFR, Beta (95% CI)		eGFR categories, Beta (95% CI)				
			Per SD Decrease	P	G2	G3a	G3b	G4 & G5	P
**Global cognition change (3MS)**	5351	14495	-0.046 (-0.073, -0.019)	<0.001[b]	0 [Ref]	-0.101 (-0.164, -0.038)	-0.156 (-0.207, -0.105)	-0.134 (-0.205, -0.064)	<0.001[b]
**Verbal memory / delayed recall (Buschke)**	3453	6364	-0.019 (-0.06, 0.023)	0.372	0 [Ref]	-0.071 (-0.173, 0.031)	-0.045 (-0.126, 0.036)	-0.015 (-0.131, 0.101)	0.403
**Attention/processing speed (Trails A)**	3496	6545	0.091 (0.053, 0.129)	<0.001[b]	0 [Ref]	0.144 (0.047, 0.241)	0.212 (0.137, 0.288)	0.265 (0.156, 0.374)	<0.001[b]
**Executive function (Trails B)**	3446	6423	0.107 (0.068, 0.146)	<0.001[b]	0 [Ref]	0.042 (-0.057, 0.140)	0.145 (0.068, 0.222)	0.259 (0.148, 0.371)	<0.001[b]

CI=Confidence interval; eGFR=estimated glomerular filtration rate; G1&G2=eGFR≥60; G3a=60>eGFR≥45; G3b=45>eGFR≥30; G4&-G5=eGFR<30; Ref=Reference category.
[a]Multivariable model includes age, sex, race and ethnicity, clincial center, education, baseline cognitive score, smoking, depression, alcohol use, physical activity, body mass index, systolic blood pressure, renin angiotensin aldosterone inhibitor use, baseline diabetes, baseline cardiovascular disease, white blood cell count, and hemoglobin.
[b]Significant after FDR correction for testing multiple cognitive endpoints

© 2026 Huang Z et al. *JAMA Network Open.*

eTable 3. Associations of CKD Based eGFR and Proteinuria With Cognitive Impairment in Fully Adjusted Models (Model 3) That Also Includes Both Kidney Function Variables[a]

	Impairment in Global Cognition (3MS)			Impairment in verbal memory/delayed recall (Buschke)			Impairment in attention/ processing speed (Trails A)			Impairment in executive function (Trails B)		
	HR	(95% CI)	P	HR	(95% CI)	P	HR	(95% CI)	P	HR	(95% CI)	P
Continuous eGFR (per SD decrease)	1.09	(0.96, 1.23)	0.177	1.09	(0.95, 1.25)	0.223	1.07	(0.92, 1.25)	0.381	1.00	(0.89, 1.13)	0.981
eGFR category												
G1&G2: >60mL/min /1.73 m^2	Ref			Ref			Ref		0.369[c]	Ref		0.848[c]
G3a: 45-59 mL/min/ 1.73 m2	1.23	(0.96, 1.58)	0.571[c]	1.32	(0.97, 1.80)	0.396[c]	0.87	(0.61, 1.25)		0.89	(0.68, 1.17)	
G3b: 30-44 mL/min/ 1.73 m2	1.09	(0.83, 1.43)		1.31	(0.93, 1.86)		1.08	(0.74, 1.56)		1.14	(0.86, 1.52)	
G4&G5:<30mL/min/ 1.73 m2	1.16	(0.82, 1.63)		1.14	(0.74, 1.76)		1.18	(0.75,1.84)		0.90	(0.62, 1.31)	
Log-UPCR (per SD Increase)	1.06	(0.95, 1.18)	0.315	1.07	(0.92, 1.25)	0.366	1.19	(1.02, 1.40)	**0.033**	1.15	(1.01, 1.32)	**0.036**
UPCR category												
P1: <150 mg/g	Ref		0.172[c]	Ref		0.465[c]	Ref		0.093[c]	Ref		0.146[c]
P2: 15-500 mg/g	1.16	(0.92, 1.46)		1.17	(0.88, 1.57)		1.40	(1.02, 1.92)		1.33	(1.03, 1.72)	
P3: >500 mg/g	1.18	(0.92, 1.51)		1.11	(0.80, 1.55)		1.31	(0.91, 1.87)		1.18	(0.88, 1.59)	
**Interaction P-value[b]**		0.141 / 0.523			0.353 / 0.292			0.281 / 0.669			0.279/ 0.986	

3MS=Modified mini-mental status exam; CI=Confidence interval; eGFR=estimated glomerular filtration rate; HR=Hazard ratio; Ref=Reference category.
[a]Multivariable model includes age, sex, race and ethnicity, clincial center, education, baseline cognitive score, smoking, depression, alcohol use, physical activity, body mass index, systolic blood pressure, renin angiotensin aldosterone inhibitor use, baseline diabetes, baseline cardiovascular disease, white blood cell count, and hemoglobin.
[b]P-value from model of continuous / ordinal eGFR and UPCR plus an interaction term for the two variables.
[c]P-value for linear trend
[d]Significant after FDR correction for testing multiple cognitive endpoints

© 2026 Huang Z et al. *JAMA Network Open.*

eTable 4. Associations of Proteinuria With Longitudinal Changes in Cognitive Tests in Fully Adjusted Models (Model 3) That Using Latent Mixed Model With Beta Link Function[a]

	No. of subject	No. of observations	Continuous Log-UPCR, Beta (95% CI)		UPCR categories			
			Per SD Increase	P	<150 mg/g	150-500 mg/g	>500 mg/g	P
**Global cognition change (3MS)**	5260	13536	-0.048 (-0.079, -0.018)	0.002[b]	0 [Ref]	-0.063 (-0.123, -0.004)	-0.088 (-0.132, -0.045)	0.022[b]
**Verbal memory /delayed recall (Buschke)**	3310	5931	0.012 (-0.040, 0.064)	0.647	0 [Ref]	-0.063 (-0.123, -0.004)	-0.034 (-0.127, 0.059)	0.814
**Attention/processing speed (Trails A)**	3364	6128	0.064 (0.016, 0.113)	0.010[b]	0 [Ref]	-0.063 (-0.123, -0.004)	0.122 (0.055, 0.189)	0.075
**Executive function (Trails B)**	3317	6015	0.082 (0.032, 0.131)	0.001[b]	0 [Ref]	-0.063 (-0.123, -0.004)	0.160 (0.091, 0.228)	0.011[b]

CI=Confidence interval; eGFR=estimated glomerular filtration rate; Ref=Reference category.
[a]Multivariable model includes age, sex, race and ethnicity, clincial center, education, baseline cognitive score, smoking, depression, alcohol use, physical activity, body mass index, systolic blood pressure, renin angiotensin aldosterone inhibitor use, baseline diabetes, baseline cardiovascular disease, white blood cell count, and hemoglobin.
[b]Significant after FDR correction for testing multiple cognitive endpoints

© 2026 Huang Z et al. *JAMA Network Open.*

eTable 5. Joint Prospective Associations Between eGFR-Based CKD and Proteinuria With Cognitive Impairment

	Joint eGFR and UPCR categories													
	G1&2/P1		G1&2/P2&3				G3-G5/P1				G3-G5/P2&3			
	N	HR	N	HR	(95% CI)	P	N	HR	(95% CI)	P	N	HR	(95% CI)	P
**Impairment in global cognition (3MS)**														
Model 1	1014	Ref	418	1.36	(0.93, 1.98)	0.109	1155	1.30	(0.99, 1.70)	0.063	1480	1.69	(1.30, 2.18)	<0.001[b]
Model 2	983	Ref	399	1.38	(0.94, 2.02)	0.099	1102	1.23	(0.93, 1.62)	0.143	1446	1.63	(1.25, 2.12)	0.002[b]
Model 3	974	Ref	394	1.25	(0.84, 1.86)	0.265	1090	1.19	(0.90, 1.58)	0.229	1431	1.38	(1.05, 1.82)	0.003[b]
**Impairment in verbal memory and delayed recall (Buschke)**														
Model 1	629	Ref	224	1.36	(0.85, 2.16)	0.198	645	1.34	(0.95, 1.88)	0.094	887	1.60	(1.15, 2.22)	0.005[b]
Model 2	609	Ref	215	1.44	(0.90, 2.31)	0.132	619	1.41	(0.99, 2.00)	0.057	861	1.64	(1.16, 2.31)	0.005[b]
Model 3	605	Ref	214	1.28	(0.79, 2.08)	0.309	613	1.38	(0.96, 1.97)	0.079	846	1.54	(1.08, 2.19)	0.016
**Impairment in attention and processing speed (Trails A)**														
Model 1	676	Ref	251	1.34	(0.79, 2.28)	0.273	696	1.03	(0.71, 1.49)	0.877	899	1.49	(1.04, 2.12)	0.028
Model 2	655	Ref	242	1.34	(0.78, 2.31)	0.292	666	1.01	(0.68, 1.48)	0.971	874	1.49	(1.03, 2.16)	0.035
Model 3	651	Ref	238	1.32	(0.76, 2.31)	0.323	658	0.93	(0.63, 1.38)	0.725	859	1.38	(0.94, 2.02)	0.103
**Impairment in executive function (Trails B)**														
Model 1	641	Ref	224	1.30	(0.88, 1.93)	0.194	629	0.96	(0.72, 1.27)	0.772	785	1.35	(1.03, 1.77)	0.032
Model 2	623	Ref	216	1.33	(0.89, 2.00)	0.170	607	0.96	(0.71, 1.29)	0.764	762	1.32	(0.99, 1.76)	0.060
Model 3	619	Ref	213	1.24	(0.82, 1.88)	0.311	600	0.96	(0.71, 1.29)	0.774	747	1.24	(0.92, 1.68)	0.164

3MS=Modified mini-mental status exam; CI=Confidence interval; eGFR=estimated glomerular filtration rate; G1&G2=eGFR≥60; G3-G5=eGFR<60; HR=Hazard ratio; P1=UPCR<150; P2&P3=UPCR≥150; Ref=Reference category.
Model 1 adjusted for age, sex, race and ethnicity, clinical center, education, and baseline cognitive score.
Model 2 adjusted for all covariables in Model 1 plus smoking, depression, alcohol use, physical activity, and body mass index.
Model 3 adjusted for all covariables in Model 2 plus systolic blood pressure, renin angiotensin aldosterone inhibitor use, baseline diabetes, baseline cardiovascular disease, white blood cell counts, and hemoglobin.
[a]P-value for any difference between categories.
[b]Significant after FDR correction for testing multiple cognitive endpoints

© 2026 Huang Z et al. *JAMA Network Open.*

eTable 6. Associations of CKD Based eGFR With Cognitive Impairment In Fully Adjusted Models (Model 3) Comparing eGFR as Estimated by the Race-Neutral, Creatinine and Cystatin C-Based CKD-Epi and CRIC Equations[a]

Equation to estimate eGFR-based CKD stage	No. of event	No. of participants	Continuous eGFR, HR (95% CI)		eGFR category, HR (95% CI)				
			Per SD Decrease	P	G1&G2	G3a	G3b	G4 & G5	$P_{Linear\ trend}$
**Impairment in global cognition (3MS)**									
CKD-EPI	529	4058	1.11 (1.00, 1.24)	0.058	1 [Ref]	1.26 (0.99, 1.61)	1.16 (0.90, 1.49)	1.26 (0.91, 1.74)	0.180
CRIC	530	4063	1.12 (1.01, 1.26)	0.041	1 [Ref]	1.21 (0.91, 1.62)	1.34 (1.00,1.79)	1.39 (0.98, 1.97)	0.040
**Impairment in verbal memory and delayed recall (Buschke)**									
CKD-EPI	349	2598	1.08 (0.97, 1.21)	0.173	1 [Ref]	1.25 (0.93, 1.67)	1.34 (0.98, 1.84)	1.21 (0.84, 1.74)	0.180
CRIC	357	2655	1.13 (0.98, 1.29)	0.090	1 [Ref]	1.00 (0.73,1.37)	1.33 (0.96, 1.85)	1.21 (0.79, 1.85)	0.090
**Impairment in attention and processing speed (Trails A)**									
CKD-EPI	295	2724	1.21 (1.05, 1.38)	0.006[b]	1 [Ref]	0.95 (0.67, 1.34)	1.18 (0.83, 1.67)	1.54 (1.05, 2.27)	0.025
CRIC	300	2785	1.18 (1.01, 1.38)	0.034	1 [Ref]	0.93 (0.65, 1.34)	1.13 (0.78, 1.64)	1.43 (0.91, 2.26)	0.083
**Impairment in executive function (Trails B)**									
CKD-EPI	451	2469	1.03 (0.93, 1.15)	0.570	1 [Ref]	0.90 (0.70, 1.16)	1.13 (0.87, 1.48)	0.99 (0.71, 1.36)	0.678
CRIC	457	2525	1.08 (0.95, 1.21)	0.238	1 [Ref]	1.15 (0.87, 1.52)	1.22 (0.91, 1.65)	1.19 (0.82, 1.75)	0.234

CI=Confidence interval; eGFR=estimated glomerular filtration rate; G1&G2=eGFR≥60; G3a=60>eGFR≥45; G3b=45>eGFR≥30; G4&-G5=eGFR<30; HR=Hazard ratio; Ref=Reference category.

[a]Multivariable model includes age, sex, race and ethnicity, clincial center, education, baseline cognitive score, smoking, depression, alcohol use, physical activity, body mass index, systolic blood pressure, renin angiotensin aldosterone inhibitor use, baseline diabetes, baseline cardiovascular disease, white blood cell count, and hemoglobin.

[b]Significant after FDR correction for testing multiple cognitive endpoints

© 2026 Huang Z et al. *JAMA Network Open.*

eTable 7. Associations of Proteinuria Compared to Albuminuria With Cognitive Impairment in Fully Adjusted Models (Model 3)[a]

	No. of event	No. of participants	Continuous Log-UPCR or Log-UACR, HR (95% CI)		UPCR or UACR categories, HR (95% CI)			
			Per SD Increase	P	P1/A1	P2/A2	P3/A3	$P_{\text{Linear trend}}$
**Impairment in global cognition (3MS)**								
UPCR	507	3920	1.09 (0.98, 1.20)	0.104	1 [Ref]	1.19 (0.95, 1.50)	1.26 (0.99, 1.59)	0.044
UACR	391	2858	1.11 (0.98, 1.25)	0.100	1 [Ref]	0.92 (0.71, 1.21)	1.27 (0.97, 1.69)	0.119
**Impairment in verbal memory and delayed recall (Buschke)**								
UPCR	317	2352	1.13 (0.99, 1.29)	0.075	1 [Ref]	1.24 (0.94, 1.64)	1.26 (0.94, 1.70)	0.103
UACR[b]		102	NA	NA	1 [Ref]	NA	NA	NA
**Impairment in attention and processing speed (Trails A)**								
UPCR	269	2486	1.21 (1.05, 1.41)	0.010[c]	1 [Ref]	1.39 (1.02, 1.89)	1.33 (0.96, 1.84)	0.053
UACR[b]		108	NA	NA	1 [Ref]	NA	NA	NA
**Impairment in executive function (Trails B)**								
UPCR	405	2252	1.16 (1.02, 1.31)	0.021[c]	1 [Ref]	1.31 (1.02, 1.68)	1.19 (0.90, 1.57)	0.122
UACR[b]		100	NA	NA	1 [Ref]	NA	NA	NA

A1=UACR<30; A2=30≤UACR<300; A3: UACR≥300; CI=Confidence interval; eGFR=estimated glomerular filtration rate; HR=Hazard ratio; P1=UPCR<150; P2=150≤UPCR≤500; P3=UPCR>500; UACR=Urinary albumin to creatinine ratio; UPCR=Urinary protein to creatinine ratio; Ref=Reference category.

[a]Multivariable model includes age, sex, race and ethnicity, clincial center, education, baseline cognitive score, smoking, depression, alcohol use, physical activity, body mass index, systolic blood pressure, renin angiotensin aldosterone inhibitor use, baseline diabetes, baseline cardiovascular disease, white blood cell count, and hemoglobin.

[b]Because the Buschke, Trails A, and Trails B tests were implemented after the CRIC baseline, when albuminuria was measured, the sample size was not sufficient to conduct these analyses.
[c]Significant after FDR correction for testing multiple cognitive endpoints

© 2026 Huang Z et al. *JAMA Network Open.*

eTable 8. Associations of CKD Based eGFR With Cognitive Impairment in Fully Adjusted Models (Model 3) That Defined Cognitive Impairment Based on a 5% Worsening of Cognition From Baseline[a]

Variable	No. of event	No. of participants	Continuous eGFR, HR (95% CI)		eGFR categories, HR (95% CI)				
			Per SD Decrease	P	G2	G3a	G3b	G4 & G5	$P_{Linear\ trend}$
**Impairment in global cognition (3MS)**									
Cutpoint of 1 SD worse than baseline mean	529	4058	1.11 (1.00, 1.24)	0.058	1 [Ref]	1.26 (0.99, 1.61)	1.16 (0.90, 1.49)	1.26 (0.91, 1.74)	0.180
Cutpoint <80	446	4159	1.12 (1.00, 1.27)	0.057	1 [Ref]	1.29 (0.99, 1.68)	1.12 (0.85, 1.49)	1.27 (0.90,1.78)	0.263
5% worsening	1413	5195	1.07 (1.01, 1.14)	0.033	1 [Ref]	1.21 (1.05, 1.40)	1.22 (1.05, 1.42)	1.14 (0.94,1.39)	0.030
**Impairment in verbal memory and delayed recall (Buschke)**									
Cutpoint of 1 SD worse than baseline mean	349	2598	1.08 (0.97, 1.21)	0.173	1 [Ref]	1.25 (0.93, 1.67)	1.34 (0.98, 1.84)	1.21 (0.84, 1.74)	0.180
5% worsening	1064	3557	1.06 (0.99, 1.12)	0.097	1 [Ref]	1.09 (0.93,1.29)	1.12 (0.94, 1.34)	1.18 (0.97,1.45)	0.090
**Impairment in attention and processing speed (Trails A)**									
Cutpoint of 1 SD worse than baseline mean	295	2724	1.21 (1.05, 1.38)	0.006[b]	1 [Ref]	0.95 (0.67, 1.34)	1.18 (0.83, 1.67)	1.54 (1.05, 2.27)	0.025
5% worsening	896	3580	1.13 (1.05, 1.22)	0.001[b]	1 [Ref]	1.05 (0.87,1.26)	1.21 (1.00, 1.47)	1.38 (1.11, 1.72)	0.002[b]
**Impairment in executive function (Trails B)**									
Cutpoint of 1 SD worse than baseline mean	451	2469	1.03 (0.93, 1.15)	0.570	1 [Ref]	0.90 (0.70, 1.16)	1.13 (0.87, 1.48)	0.99 (0.71, 1.36)	0.678
5% worsening	1581	3541	1.05 (1.00, 1.11)	0.069	1 [Ref]	1.07 (0.94, 1.22)	1.13 (0.98, 1.31)	1.05 (0.88, 1.25)	0.275

CI=Confidence interval; eGFR=estimated glomerular filtration rate; G1&G2=eGFR≥60; G3a=60>eGFR≥45; G3b=45>eGFR≥30; G4&-G5=eGFR<30; HR=Hazard ratio; Ref=Reference category.

[a]Multivariable model includes age, sex, race and ethnicity, clincial center, education, baseline cognitive score, smoking, depression, alcohol use, physical activity, body mass index, systolic blood pressure, renin angiotensin aldosterone inhibitor use, baseline diabetes, baseline cardiovascular disease, white blood cell count, and hemoglobin.

[b]Significant after FDR correction for testing multiple cognitive endpoints

© 2026 Huang Z et al. *JAMA Network Open.*

eTable 9. Associations of Proteinuria With Cognitive Impairment in Fully Adjusted Models (Model 3) That Defined Cognitive Impairment as a 5% Worsening Since Baseline[a]

	No. of event	No. of participants	Continuous Log-UPCR		UPCR categories			
			Per SD Increase	P	<150 mg/g	150-500 mg/g	>500 mg/g	$P_{\text{Linear trend}}$
**Impairment in global cognition (3MS)**								
Cutpoint of 1 SD worse than baseline mean	507	3920	1.09 (0.98, 1.20)	0.104	1 [Ref]	1.19 (0.95, 1.50)	1.26 (0.99, 1.59)	0.044
Cutpoint <80	428	3985	1.06 (0.95, 1.18)	0.318	1 [Ref]	1.14 (0.89, 1.46)	1.20 (0.93, 1.55)	0.153
5% worsening	1352	4941	1.07 (1.01, 1.14)	0.032[b]	1 [Ref]	1.15 (1.00, 1.32)	1.17 (1.01,1.35)	0.024[b]
**Impairment in verbal memory and delayed recall (Buschke)**								
Cutpoint of 1 SD worse than baseline mean	317	2352	1.13 (0.99, 1.29)	0.075	1 [Ref]	1.24 (0.94, 1.64)	1.26 (0.94, 1.70)	0.103
5% worsening	954	3177	1.05 (0.97, 1.14)	0.205	1 [Ref]	1.09 (0.93, 1.29)	1.06 (0.89, 1.27)	0.412
**Impairment in attention and processing speed (Trails A)**								
Cutpoint of 1 SD worse than baseline mean	269	2486	1.21 (1.05, 1.41)	0.010[b]	1 [Ref]	1.39 (1.02, 1.89)	1.33 (0.96, 1.84)	0.053
5% worsening	815	3202	1.15 (1.06, 1.26)	0.002[b]	1 [Ref]	1.16 (0.97, 1.39)	1.32 (1.09, 1.59)	0.004[b]
**Impairment in executive function (Trails B)**								
Cutpoint of 1 SD worse than baseline mean	405	2252	1.16 (1.02, 1.31)	0.021[b]	1 [Ref]	1.31 (1.02, 1.68)	1.19 (0.90, 1.57)	0.122
5% worsening	1433	3167	1.09 (1.03, 1.17)	0.007[b]	1 [Ref]	1.18 (1.03,1.34)	1.14 (0.98, 1.32)	0.034[b]

CI=Confidence interval; eGFR=estimated glomerular filtration rate; HR=Hazard ratio; Ref=Reference category.

[a]Multivariable model includes age, sex, race and ethnicity, clincial center, education, baseline cognitive score, smoking, depression, alcohol use, physical activity, body mass index, systolic blood pressure, renin angiotensin aldosterone inhibitor use, baseline diabetes, baseline cardiovascular disease, white blood cell count, and hemoglobin.

[b]Significant after FDR correction for testing multiple cognitive endpoints

© 2026 Huang Z et al. *JAMA Network Open.*

eTable 10. Restrict Cubic Spline of CKD Based eGFR and Proteinuria With Cognitive Impairment in Fully Adjusted Models (Model 3)[a] That Used Different Knots

Knots (N)	Global cognition (3MS)		Verbal memory/delayed recall (Buschke)		Attention/processing speed (Trails A)		Executive function (Trails B)	
	$P_{Non\text{-}linear\ trend}$	$P_{Linear\ trend}$	$P_{Non\text{-}linear\ trend}$	$P_{Linear\ trend}$	$P_{Non\text{-}linear\ trend}$	$P_{Linear\ trend}$	$P_{Non\text{-}linear\ trend}$	$P_{Linear\ trend}$
**Per standard change in eGFR**								
3	0.064		0.332		0.348		0.591	0.420
4	0.167	0.035	0.432	0.179	0.506	0.004[b]	0.857	
5	0.289		0.607		0.604		0.944	
**Log transformed UPCR**								
3	0.766		0.570		0.457		0.230	
4	0.784	0.122	0.544	0.079	0.765	0.012[b]	0.345	0.029
5	0.893		0.621		0.565		0.476	

[a]Multivariable model includes age, sex, race and ethnicity, clincial center, education, baseline cognitive score, smoking, depression, alcohol use, physical activity, body mass index, systolic blood pressure, renin angiotensin aldosterone inhibitor use, baseline diabetes, baseline cardiovascular disease, white blood cell count, and hemoglobin.
[b]Significant after FDR correction for testing multiple cognitive endpoints

© 2026 Huang Z et al. *JAMA Network Open.*

eTable 11. Associations of CKD Based eGFR With Cognitive Impairment in Fully Adjusted Models (Model 3) That With Death as Competing Risk Event[a]

Variable	No. of event	No. of participants	Continuous eGFR, HR (95% CI)		eGFR categories, HR (95% CI)				
			Per SD Decrease	P	G2	G3a	G3b	G4 & G5	$P_{Linear\ trend}$
**Impairment in global cognition (3MS)**									
Cause-specific hazard ratio	529	4058	1.11 (1.00, 1.24)	0.058	1 [Ref]	1.26 (0.99, 1.61)	1.16 (0.90, 1.49)	1.26 (0.91, 1.74)	0.180
Subdistribution hazard ratio	529	4058	1.08 (0,97, 1.21)	0.150	1 [Ref]	1.27 (0.99, 1.60)	1.12 (0.86, 1.45)	1.16 (0.84, 1.60)	0.400
**Impairment in verbal memory and delayed recall (Buschke)**									
Cause-specific hazard ratio	349	2598	1.08 (0.97, 1.21)	0.173	1 [Ref]	1.25 (0.93, 1.67)	1.34 (0.98, 1.84)	1.21 (0.84, 1.74)	0.180
Subdistribution hazard ratio	349	2598	1.07 (0.96, 1.19)	0.220	1 [Ref]	1.23 (0.93, 1.64)	1.34 (0.97, 1.83)	1.16 (0.82,1.65)	0.230
**Impairment in attention and processing speed (Trails A)**									
Cause-specific hazard ratio	295	2724	1.21 (1.05, 1.38)	0.006[b]	1 [Ref]	0.95 (0.67, 1.34)	1.18 (0.83, 1.67)	1.54 (1.05, 2.27)	0.025
Subdistribution hazard ratio	295	2724	1.19 (1.04, 1.35)	0.009[b]	1 [Ref]	0.97 (0.69, 1.35)	1.20 (0.85, 1.69)	1.52 (1.06, 2.18)	0.024
**Cognitive impairment (Trails B)**									
Cause-specific hazard ratio	451	2469	1.03 (0.93, 1.15)	0.570	1 [Ref]	0.90 (0.70, 1.16)	1.13 (0.87, 1.48)	0.99 (0.71, 1.36)	0.678
Subdistribution hazard ratio	451	2469	1.03 (0.93, 1.14)	0.600	1 [Ref]	0.91 (0.71, 1.17)	1.12 (0.87, 1.45)	0.99 (0.73, 1.35)	0.670

CI=Confidence interval; eGFR=estimated glomerular filtration rate; G1&G2=eGFR≥60; G3a=60>eGFR≥45; G3b=45>eGFR≥30; G4&-G5=eGFR<30; HR=Hazard ratio; Ref=Reference category.

[a]Multivariable model includes age, sex, race and ethnicity, clincial center, education, baseline cognitive score, smoking, depression, alcohol use, physical activity, body mass index, systolic blood pressure, renin angiotensin aldosterone inhibitor use, baseline diabetes, baseline cardiovascular disease, white blood cell count, and hemoglobin.

[b]Significant after FDR correction for testing multiple cognitive endpoints

© 2026 Huang Z et al. *JAMA Network Open.*

eTable 12. Associations of Proteinuria With Cognitive Impairment in Fully Adjusted Models (Model 3) That With Death as Competing Risk Event[a]

Variable	No. of event	No. of participants	Continuous Log-UPCR		UPCR categories			
			Per SD Increase	P	<150 mg/g	150-500 mg/g	>500 mg/g	$P_{Linear\ trend}$
**Impairment in global cognition (3MS)**								
Cause-specific hazard ratio	507	3920	1.09 (0.98, 1.20)	0.104	1 [Ref]	1.19 (0.95, 1.50)	1.26 (0.99, 1.59)	0.044
Subdistribution hazard ratio	507	3920	1.07 (0.97, 1.18)	0.200	1 [Ref]	1.15 (0.92, 1.43)	1.19 (0.94,1.51)	0.110
**Impairment in verbal memory and delayed recall (Buschke)**								
Cause-specific hazard ratio	317	2352	1.13 (0.99, 1.29)	0.075	1 [Ref]	1.24 (0.94, 1.64)	1.26 (0.94, 1.70)	0.103
Subdistribution hazard ratio	317	2352	1.12 (0.98, 1.27)	0.092	1 [Ref]	1.14 (0.87, 1.50)	1.22 (0.92, 1.61)	0.160
**Impairment in attention and processing speed (Trails A)**								
Cause-specific hazard ratio	269	2486	1.21 (1.05, 1.41)	0.010[b]	1 [Ref]	1.39 (1.02, 1.89)	1.33 (0.96, 1.84)	0.053
Subdistribution hazard ratio	269	2486	1.18 (1.02, 1.35)	0.023	1 [Ref]	1.31 (0.97, 1.76)	1.32 (0.97, 1.79)	0.051
**Cognitive impairment (Trails B)**								
Cause-specific hazard ratio	405	2252	1.16 (1.02, 1.31)	0.021[b]	1 [Ref]	1.31 (1.02, 1.68)	1.19 (0.90, 1.57)	0.122
Subdistribution hazard ratio	405	2252	1.12 (0.99, 1.27)	0.071	1 [Ref]	1.28 (1.00, 1.64)	1.18 (0.89, 1.55)	0.140

CI=Confidence interval; eGFR=estimated glomerular filtration rate; HR=Hazard ratio; Ref=Reference category.
[a]Multivariable model includes age, sex, race and ethnicity, clincial center, education, baseline cognitive score, smoking, depression, alcohol use, physical activity, body mass index, systolic blood pressure, renin angiotensin aldosterone inhibitor use, baseline diabetes, baseline cardiovascular disease, white blood cell count, and hemoglobin.
[b]Significant after FDR correction for testing multiple cognitive endpoints

© 2026 Huang Z et al. *JAMA Network Open.*

eFigure 1. Restricted Cubic Spline Plots of Estimated Glomerular Filtration Rate

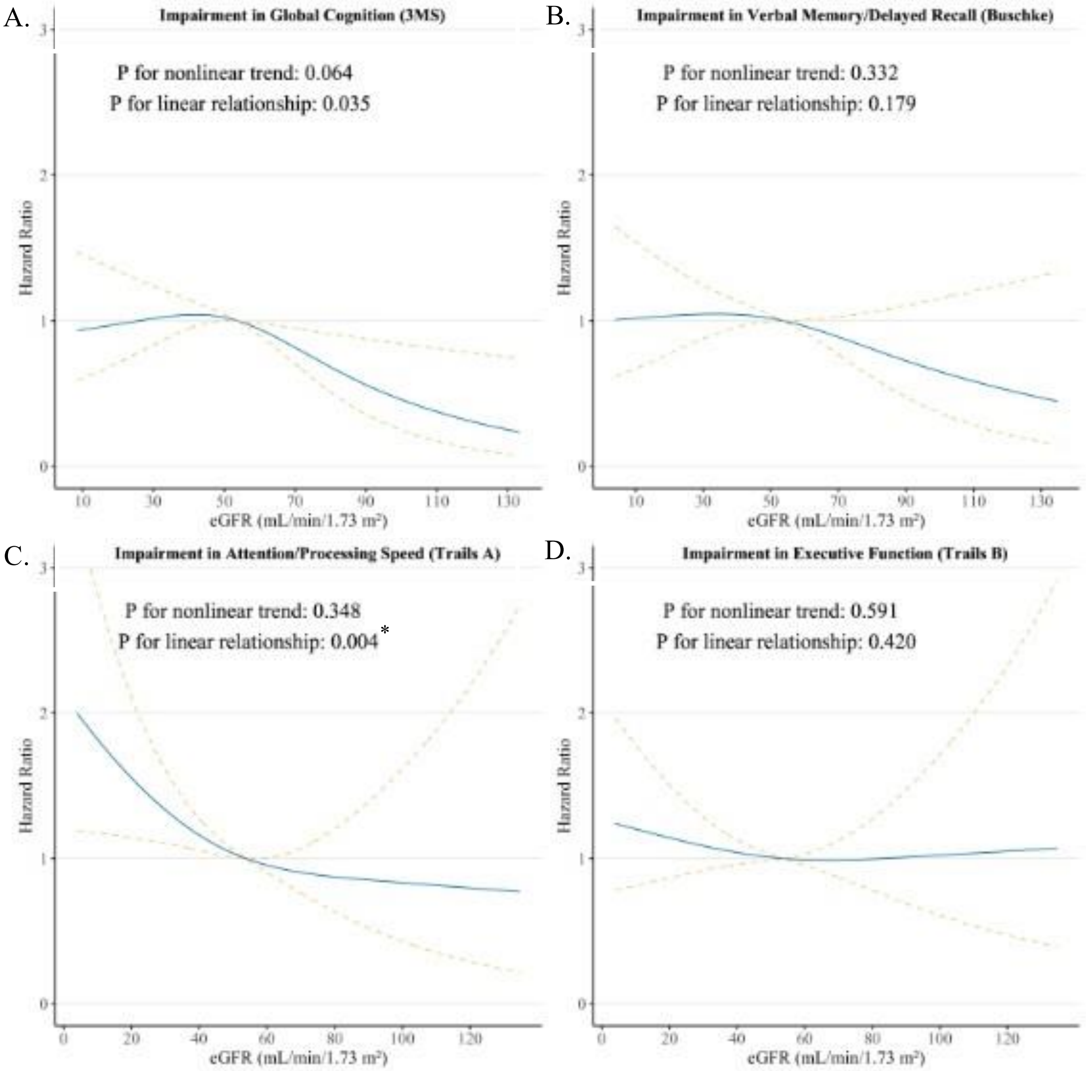

* Significant after FDR correction for testing multiple cognitive endpoints

Figure legend: Restricted cubic spline plots of multivariable hazard ratios corresponding to each unit increase in estimated glomerular filtration rate for cognitive impairment based on the: A) Modified Mini-Mental State Examination, B) Buschke Selective Reminding Test, C) Trail Making Test A, and D) Trail Making Test B.

© 2026 Huang Z et al. *JAMA Network Open.*

eFigure 2. Restricted Cubic Spline Plots of Log-Transformed Urinary Protein-to-Creatinine Ratio

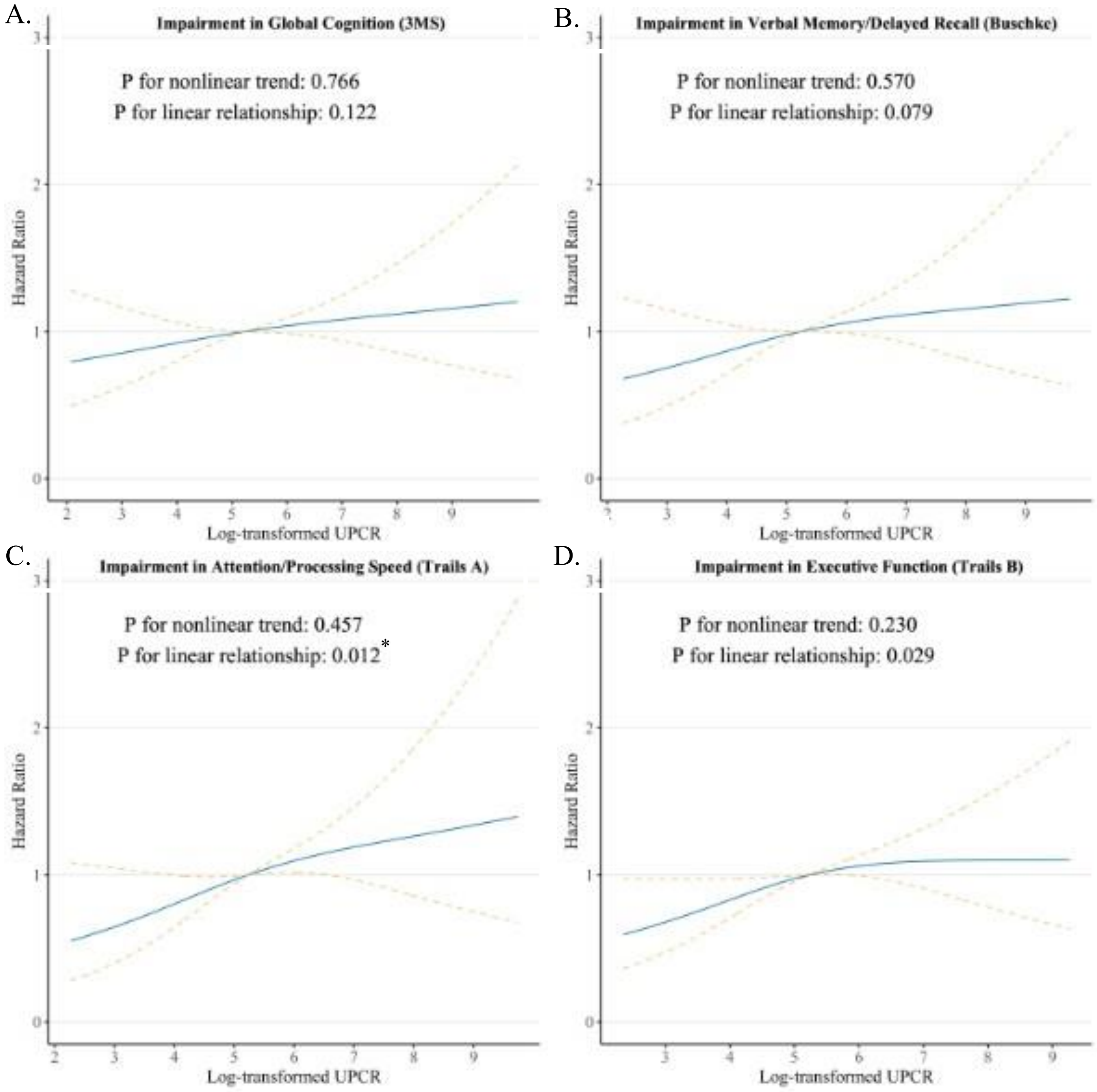

* Significant after FDR correction for testing multiple cognitive endpoints

Figure legend: Restricted cubic spline plots of multivariable hazard ratios corresponding to each unit increase in log-transformed urinary protein-to-creatinine ratio for cognitive impairment based on the: A) Modified Mini-Mental State Examination, B) Buschke Selective Reminding Test, C) Trail Making Test A, and D) Trail Making Test B.

© 2026 Huang Z et al. *JAMA Network Open.*

eFigure 3. Multivariable Adjusted Hazard Ratios for Cognitive Impairment Corresponding to Each Standard Deviation Decrease in eGFR

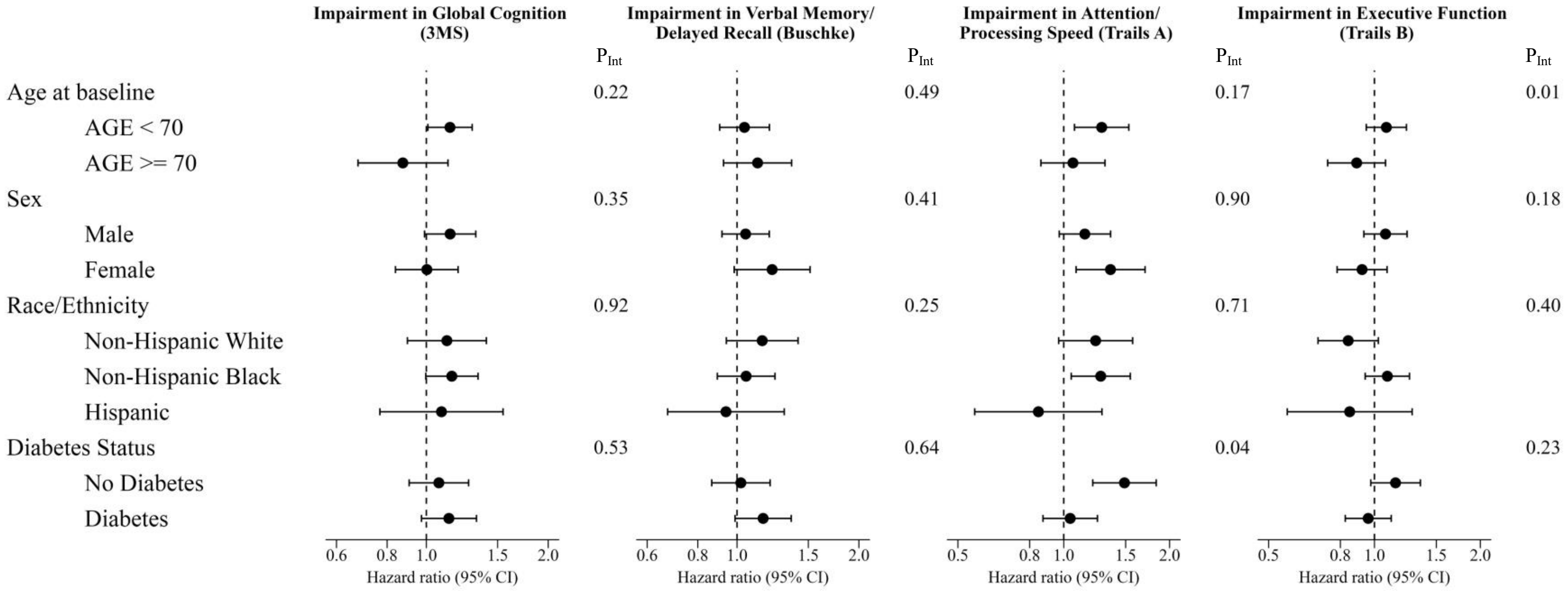

© 2026 Huang Z et al. *JAMA Network Open.*

eFigure 4. Multivariable Adjusted Hazard Ratios for Cognitive Impairment Corresponding to Each Standard Deviation Increase in UPCR

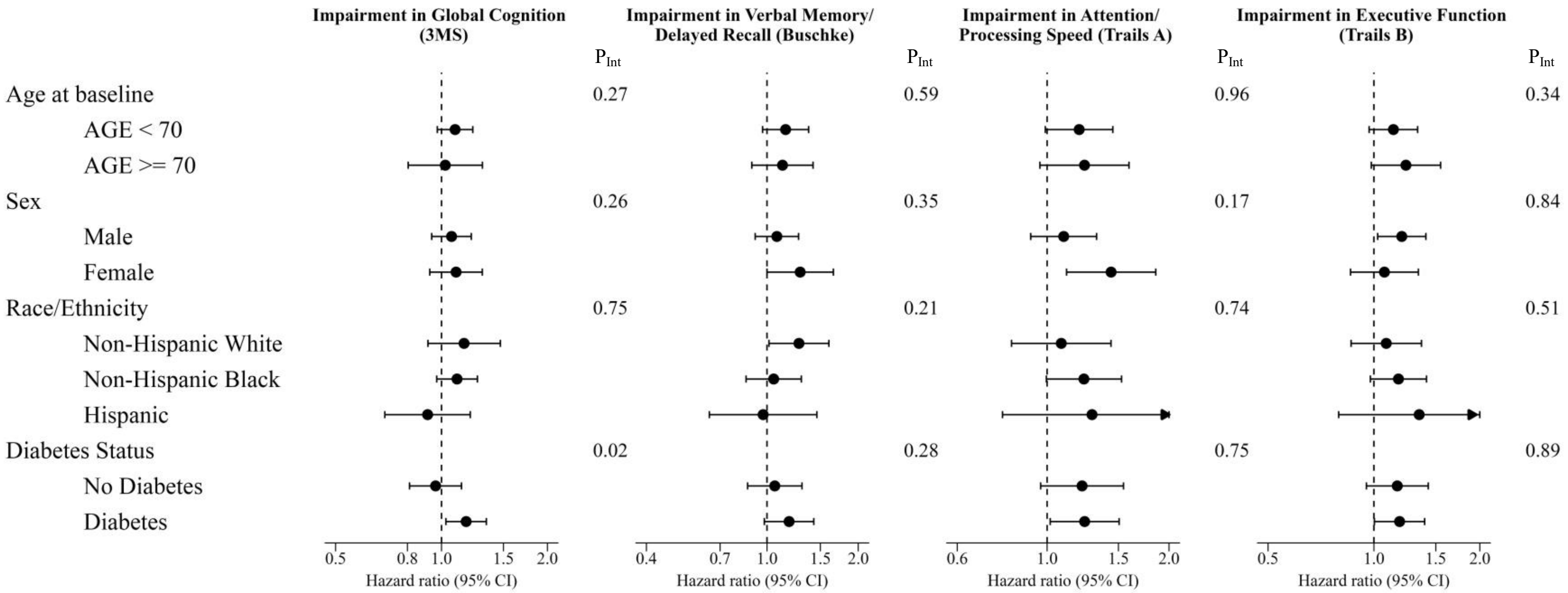

© 2026 Huang Z et al. *JAMA Network Open.*

eMethods.

CRIC Study Population

CRIC is an on-going prospective cohort study that aims to identify novel risk factors for the progression of CKD and cardiovascular disease (CVD) in the setting of CKD[1]. Since the start of recruitment in 2003, CRIC has enrolled 5,607 participants with CKD at seven clinical centers. CRIC recruited a racially and ethnically diverse sample of adults ranging from 21 to 79 years of age with a broad spectrum of kidney disease severity (eGFR of 20-70 ml/min/1.73 m^2). Among these participants, about half of them had diabetes, and 19% had advanced CKD (eGFR<30 mL/min/1.73 m^2) at baseline. Exclusion criteria included polycystic kidney disease, recent immunosuppression for renal disease, previous dialysis for >1-month, current pregnancy, and inability to give informed consent. After excluding participants with baseline cognitive impairment or missing baseline kidney function data or at least one repeated measure of cognition, a total of 4261, 2726, 2866, and 2591 participants were included in analyses of cognitive impairment as assessed by the Modified Mini-Mental State Examination (3MS), Buschke Selective Reminding Test (Buschke), and Trail Making Tests A and B (Trails A and B, respectively), with median (minimum, maximum) follow-up years of 6 (0.5, 16), 4 (0.5, 13), 4 (0.5, 13), and 4 (0.5, 13), respectively.

Exposure Assessment

Serum creatinine was measured using an enzymatic method (Ortho Clinical Diagnostics) until October 2008 and the Jaffe method (Beckman Coulter), with values calibrated to account for variability across laboratories and time[2]. Cystatin C was measured using a particle-enhanced immunophelometric assay.

Covariable measures

Body mass index (BMI) was calculated as mean weight in kilograms divided by mean height in meters squared. Hypertension was defined as a systolic BP≥130 mmHg, diastolic BP≥80 mmHg, or use of blood pressure lowering medication. Standard laboratory assays were used to measure glucose, white blood cell counts, and hemoglobin. Diabetes was defined as a fasting glucose ≥126 mg/dL, non-fasting glucose ≥200 mg/dL, or the use of glucose lowering medications.

Cognitive function assessment

Global cognitive function has been assessed annually or biennially since 2003 using the 3MS, which incorporates components of concentration, orientation, language, and memory[3]. 3MS scores range from 0 to 100, and higher scores indicate better cognitive function. Introduced in 2006 at four of the seven CRIC clinical centers and extended to all participants by 2008, the Buschke, Trails A, and Trails B tests were administered annually or biennially. The Buschke evaluates verbal memory/delayed recall[4], with scores ranging from 0 to 12 and higher scores indicating better cognitive function. Trails A and B evaluate domains of attention/processing speed and executive function[5], respectively, with scores ranging from 0 to 300 and higher scores indicating poorer cognitive function. To align our analyses with previous reports from the CRIC study[6–8], incident cognitive impairment was defined for each test as a score at least one standard deviation (SD) worse than the cohort mean at baseline. In all longitudinal analyses, those with cognitive impairment at baseline were excluded. To assess the robustness of our findings to alternate definitions of cognitive impairment, we further defined cognitive impairment as a 3MS score <80, which has been employed in several previous reports[9,10], and by a test score at least 5% worse than the baseline score, which reflects meaningful cognitive change[11].

Statistical analysis

After excluding those with cognitive impairment at baseline, associations of eGFR, UPCR, and joint eGFR/UPCR with incident cognitive impairment were assessed using three Cox proportional hazards models. Model 1 adjusted for demographic variables (age, sex, race/ethnicity, and educational attainment), along with clinical center and baseline cognitive score. Model 2 additionally adjusted for lifestyle and behavioral risk factors including smoking, alcohol drinking, physical activity, depression, and BMI. The fully adjusted model, Model 3, further included clinical variables such as systolic blood pressure, diabetes, cardiovascular disease history, use of angiotensin converting enzyme inhibitors or angiotensin receptor blockers, white blood cell count, and hemoglobin.

To evaluate associations of eGFR and UPCR with longitudinal changes in cognitive test scores, we also implemented latent process mixed models with a beta link for each cognitive test. These models account for the discrete and curvilinearity properties of psychometric tests, including ceiling and floor effects, in longitudinal studies of cognitive aging[12–14]. The latent process represents the underlying unobserved cognitive level, whose change over time is modeled using a linear mixed model with covariates, while an observation equation links the latent process to the observed test scores. The beta linear mixed model, as proposed by Proust et al.[15], was selected because it was found to have a fit comparable to threshold mixed models and to provide unbiased inference in simulations, while avoiding substantial computational complexity[16].

© 2026 Huang Z et al. *JAMA Network Open.*

eReferences.

1. Lash JP, Go AS, Appel LJ, et al. Chronic Renal Insufficiency Cohort (CRIC) Study: Baseline Characteristics and Associations with Kidney Function. *Clin J Am Soc Nephrol*. 2009;4(8):1302. doi:10.2215/CJN.00070109

2. Joffe M, Hsu CY, Feldman HI, Weir M, Landis JR, Hamm LL. Variability of creatinine measurements in clinical laboratories: results from the CRIC study. *Am J Nephrol*. 2010;31(5):426-434. doi:10.1159/000296250

3. Chui TEL. Modified Mini Mental State Examination (3MS).

4. Buschke H, Fuld PA. Evaluating storage, retention, and retrieval in disordered memory and learning. *Neurology*. 1974;24(11):1019-1025. doi:10.1212/WNL.24.11.1019

5. Llinàs-Reglà J, Vilalta-Franch J, López-Pousa S, Calvó-Perxas L, Torrents Rodas D, Garre-Olmo J. The Trail Making Test. *Assessment*. 2017;24(2):183-196. doi:10.1177/1073191115602552

6. Xiao C, Tamura MK, Pan Y, et al. Clonal hematopoiesis of indeterminate potential is associated with reduced risk of cognitive impairment in patients with chronic kidney disease. *Alzheimer's & Dementia*. 2024;20(10):6960-6971. doi:10.1002/ALZ.14182

7. Babroudi S, Tighiouart H, Schrauben SJ, et al. Blood Pressure, Incident Cognitive Impairment, and Severity of CKD: Findings From the Chronic Renal Insufficiency Cohort (CRIC) Study. *American Journal of Kidney Diseases*. 2023;82(4):443-453.e1. doi:10.1053/j.ajkd.2023.03.012

8. Kurella Tamura M, Tam K, Vittinghoff E, et al. Inflammatory Markers and Risk for Cognitive Decline in Chronic Kidney Disease: The CRIC Study. *Kidney Int Rep*. 2017;2(2):192-200. doi:10.1016/J.EKIR.2016.10.007

9. Lin FR, Yaffe K, Xia J, et al. Hearing Loss and Cognitive Decline in Older Adults. *JAMA Intern Med*. 2013;173(4):293. doi:10.1001/jamainternmed.2013.1868

10. Bernick C, Katz R, Smith NL, et al. Statins and cognitive function in the elderly. *Neurology*. 2005;65(9):1388-1394. doi:10.1212/01.wnl.0000182897.18229.ec

11. Lidgard B, Bansal N, Zelnick LR, et al. Association of Proximal Tubular Secretory Clearance with Long-Term Decline in Cognitive Function. *Journal of the American Society of Nephrology*. 2022;33(7):1391-1401. doi:10.1681/ASN.2021111435

12. Ganiayre J, Commenges D, Letenneur L. A latent process model for dementia and psychometric tests. *Lifetime Data Anal*. 2008;14(2):115-133. doi:10.1007/s10985-007-9057-x

13. Proust C, Jacqmin-Gadda H, Taylor JMG, Ganiayre J, Commenges D. A Nonlinear Model with Latent Process for Cognitive Evolution Using Multivariate Longitudinal Data. *Biometrics*. 2006;62(4):1014-1024. doi:10.1111/J.1541-0420.2006.00573.X

14. Jacqmin-Gadda H, Proust-Lima C, Amiéva H. Semi-parametric latent process model for longitudinal ordinal data: Application to cognitive decline. *Stat Med*. 2010;29(26):2723-2731. doi:10.1002/sim.4035

15. Proust C, Jacqmin-Gadda H, Taylor JMG, Ganiayre J, Commenges D. A Nonlinear Model with Latent Process for Cognitive Evolution Using Multivariate Longitudinal Data. *Biometrics*. 2006;62(4):1014-1024. doi:10.1111/j.1541-0420.2006.00573.x

16. Proust-Lima C, Dartigues JF, Jacqmin-Gadda H. Misuse of the linear mixed model when evaluating risk factors of cognitive decline. *Am J Epidemiol*. 2011;174(9):1077. doi:10.1093/AJE/KWR243

© 2026 Huang Z et al. *JAMA Network Open.*
