# Supplementary material for: Chronic Kidney Disease Severity and Risk of Cognitive Impairment
Source: JAMA Netw Open. 2026 Feb 17;9(2):e2559834. doi: 10.1001/jamanetworkopen.2025.59834 (PMC12914485; doi:10.1001/jamanetworkopen.2025.59834)
Supplement: Supplement 2. — Nonauthor Collaborators. Chronic Renal Insufficiency Cohort (CRIC) Study Investigators [file jamanetwopen-e2559834-s002.pdf]

Supplemental Online Content: Nonauthor Collaborators
*First name, last name, and suffix (if applicable) are required and will appear in PubMed.

*Group Name(s): Chronic Renal Insufficiency Cohort (CRIC) Study Investigators							
***First Name and Middle Initial(s)**	***Last Name**	***Suffix (eg, Jr, III)**	Academic Degrees	Institution	Location (city, state/province, country)	Role or Contribution, eg, chair, principal investigator	Group (if more than 1 Group listed in the byline) and/or Subgroup (eg, Steering Committee)
Amanda H.	Anderson		PhD, MPH	Department of Epidemiology, University of Alabama at Birmingham School of Public Health	Birmingham, Alabama, USA	principal investigator	
Lawrence J.	Appel		MD, MPH	Division of General Internal Medicine, Johns Hopkins School of Medicine	Baltimore, Maryland, USA	principal investigator	
Jing	Chen		MD, MMSc, MSc	Department of Epidemiology, Peter O'Donnell Jr. School of Public Health at UT Southwestern Medical Center	Dallas, Texas, USA	principal investigator	
Debbie L.	Cohen		MD	Division of Renal, Electrolyte and Hypertension, Hospital of the University of Pennsylvania	Philadelphia, Pennsylvania, USA	principal investigator	
Laura M.	Dember		MD	Department of Biostatistics, Epidemiology and Informatics, Perelman School of Medicine, University of Pennsylvania	Philadelphia, Pennsylvania, USA	principal investigator	
Alan S.	Go		MD	Division of Research, Kaiser Permanente Northern California	Pleasanton, California, USA	principal investigator	
James P.	Lash		MD	Division of Nephrology, Department of Medicine, University of Illinois College of Medicine	Chicago, Illinois, USA	principal investigator	
Mahboob	Rahman		MD	Division of Nephrology and Hypertension, University Hospitals Cleveland Medical Center	Cleveland, Ohio, USA	principal investigator	

Supplemental Online Content: Nonauthor Collaborators
*First name, last name, and suffix (if applicable) are required and will appear in PubMed.

***First Name and Middle Initial(s)**	***Last Name**	***Suffix (eg, Jr, III)**	Academic Degrees	Institution	Location (city, state/province, country)	Role or Contribution, eg, chair, principal investigator	Group (if more than 1 Group listed in the byline) and/or Subgroup (eg, Steering Committee)
Panduranga S.	Rao		MD	Division of Nephrology, Department of Internal Medicine, University of New Mexico,	Albuquerque, New Mexico, USA	principal investigator	
Vallabh O.	Shah		PhD, MS	Department of Internal Medicine and Biochemistry, University of New Mexico,	Albuquerque, New Mexico, USA	principal investigator	
Mark L.	Unruh		MD, MS	Division of Nephrology, Department of Internal Medicine, University of New Mexico,	Albuquerque, New Mexico, USA	principal investigator	
