# Supplementary material for: Chronic Kidney Disease Severity and Risk of Cognitive Impairment
Source: JAMA Netw Open. 2026 Feb 17;9(2):e2559834. doi: 10.1001/jamanetworkopen.2025.59834 (PMC12914485; doi:10.1001/jamanetworkopen.2025.59834)
Supplement: Supplement 3. — Data Sharing Statement [file jamanetwopen-e2559834-s003.pdf]

Data Sharing Statement

Huang. Chronic Kidney Disease Severity and Risk of Cognitive Impairment. *JAMA Netw Open*. Published February 17, 2026. doi:10.1001/jamanetworkopen.2025.59834

Data
Data available: Yes
Data types: Deidentified participant data, Data dictionary
How to access data: Data from Chronic Renal Insufficiency Cohort Study (CRIC) [(Version 13) https://doi.org/10.58020/6dxf-ed78] reported here are available for request at the NIDDK Central Repository (NIDDK-CR) website, Resources for Research (R4R), https://repository.niddk.nih.gov/.
When available: With publication

Supporting Documents
Document types: None

Additional Information
Who can access the data: Researchers whose proposed use of the data has been approved.
Types of analyses: As specified by the proposal submitted to the NIDDK Central repository.
Mechanisms of data availability: Without investigator support and after approval of a proposal submitted to the NIDDK Central Repository.
